# Temporal Flexibility of Systems Consolidation and the Synaptic Occupancy/Reset Theory (SORT): Cues About the Nature of the Engram

**DOI:** 10.3389/fnsyn.2019.00001

**Published:** 2019-02-13

**Authors:** Jorge Alberto Quillfeldt

**Affiliations:** ^1^Psychobiology and Neurocomputation Lab, Department of Biophysics, Institute of Biosciences, Federal University of Rio Grande do Sul, Porto Alegre, Brazil; ^2^Neurosciences Graduate Program, Institute of Basic Health Sciences, Federal University of Rio Grande do Sul, Porto Alegre, Brazil; ^3^Department of Psychology, McGill University, Montreal, QC, Canada

**Keywords:** systems consolidation temporal framework, recent vs. remote memory, precision vs. generalization, hippocampus, neocortex, Synaptic Occupancy/Reset Theory (SORT)

## Abstract

The ability to adapt to new situations involves behavioral changes expressed either from an innate repertoire, or by acquiring experience through memory consolidation mechanisms, by far a much richer and flexible source of adaptation. Memory formation consists of two interrelated processes that take place at different spatial and temporal scales, *Synaptic Consolidation*, local plastic changes in the recruited neurons, and *Systems Consolidation, a* process of gradual reorganization of the explicit/declarative memory trace between hippocampus and the neocortex. In this review, we summarize some converging experimental results from our lab that support a normal temporal framework of memory systems consolidation as measured both from the anatomical and the psychological points of view, and propose a hypothetical model that explains these findings while predicting other phenomena. Then, the same experimental design was repeated interposing additional tasks between the training and the remote test to verify for any interference: we found that (a) when the animals were subject to a succession of new learnings, systems consolidation was accelerated, with the disengagement of the hippocampus taking place before the natural time point of this functional switch, but (b) when a few reactivation sessions reexposed the animal to the training context without the shock, systems consolidation was delayed, with the hippocampus prolonging its involvement in retrieval. We hypothesize that new learning recruits from a fixed number of plastic synapses in the CA1 area to store the engram index, while reconsolidation lead to a different outcome, in which additional synapses are made available. The first situation implies the need of a reset mechanism in order to free synapses needed for further learning, and explains the acceleration observed under intense learning activity, while the delay might be explained by a different process, able to generate extra free synapses: depending on the cognitive demands, it deals either with a fixed or a variable pool of available synapses. The Synaptic Occupancy/Reset Theory (SORT) emerged as an explanation for the temporal flexibility of systems consolidation, to encompass the two different dynamics of explicit memories, as well as to bridge both synaptic and systems consolidation in one single mechanism.

## Memory and Time

The ability to adapt to challenging new situations involves both physiological and behavioral changes, and behavior may either be expressed from an innate repertoire of stereotyped responses – which Fuster (1995) calls “phyletic memory” – or by the acquisition of experience through memory mechanisms, or even a combination of both (James, 1890). These two classes of cognitive functions, however, differ in several respects, and the second one – “individual memory” – is by far a much richer and flexible source of both adaptation and resilience (two complementary concepts according to Wong-Parodi et al., 2015), and, ultimately, might be the reason for the evolutionary success of vertebrates, specially the mammals.

Memory is an experience-based behavior modification. This is a purely operational definition that covers the basic types of memory that humans and non-human animals fully share, leaving imaginary and/or abstract constructions – whose relation to behavior is somewhat distant – aside for a while. In order to be preserved, it is generally accepted that this change demands the storage (and retrievability) of a physical trace that somehow embodies the experience (Craik, 2002). However, we still don’t know how much (and exactly which) information is effectively stored, with possibilities varying from a simple set of reconstruction instructions (Bartlett, 1932; Neisser, 1967; Roediger and De Soto, 2015) up to a larger collection of detailed information.

Memory formation consists of two interrelated processes, equally referred to as *consolidation*, that take place at different spatial and temporal scales. *Synaptic* (or *Cellular*) *Consolidation* comes first and consists of local plastic changes in the recruited neurons *in each and every brain area involved* in order to re-structure synaptic connections, lasting from minutes to hours (Dudai, 1996). Over a much larger time scale, *Systems Consolidation* is the process of gradual reorganization of the explicit (non-episodic-like) memory trace in the NCTX, along with progressive independence from the HPC and its adjacent cortices – which in rats takes a few weeks, but in humans can take from months to years (Kim and Fanselow, 1992; McClelland et al., 1995; Dudai, 1996; Quillfeldt et al., 1996; Izquierdo et al., 1997; Frankland and Bontempi, 2005; Winocur et al., 2010; Wiltgen et al., 2010).

Evidence for memory systems consolidation began to emerge in studies with lesioned patients already in the 19th century (e.g., Ribot, 1881), but it was only after the paradigmatic case of patient H. M. (Henry Molaisson), described by Scoville and Milner (1957), that the HPC was singled out as a crucial structure for memory (McDonald and White, 1993; Squire et al., 1984; Squire, 2004). Lesions restricted to the MTL, that includes the hippocampal formation, resulted in temporally graded RA – the loss of the memories acquired more recently with some degree of preservation of the older ones, as well as a severe anterograde amnesia – the inability to code for new long-term memories (Squire and Bayley, 2007; Nadel et al., 2007).

## Episodic Memory in Time: Clash Between Facts and Theories

Systems consolidation, with a functional “transition” between HPC and NCTX, has been mostly verified for the so-called explicit or declarative memory, which in humans involve two categories, episodic and semantic memories (Tulving, 1972; Cohen and Squire, 1980; Cohen, 1981; Graf and Schacter, 1985): however, human episodic memory have resisted to conform to this dynamics since it typically remains indefinitely dependent from the HPC – non-graded or “flat” temporal gradient RA (Nadel et al., 2007; Nadel and Hardt, 2011). Episodic memory is still at the fulcrum of a decades-old debate between two competing theories about temporal modifications undergone by explicit memories. The first, conventionally known as the SMSC (Squire and Alvarez, 1995), proposes that all long-term memories already consolidated at the synaptic level (i.e., after at least 6hs), in the beginning need the HPC to be retrieved, but this dependence will subside progressively, with memory processes becoming reliant upon neocortical circuits. SMSC holds that *all declarative* memories, be them of episodic or semantic nature, must have the same fate, becoming independent from the HPC. After an extensive review of the literature on human memory, however, it became clear that the remote episodic memories cannot usually be retrieved without the assistance of the HPC (Nadel and Moscovitch, 1997), which paved the way for the more encompassing conception known as the MTT, which accepts, among other concepts, different dynamics for episodic and semantic memories.

For a number of reasons, it was not trivial to reproduce, in animal models, the clear-cut division between episodic and semantic memories observed in humans, but, similar to humans, the temporally graded RA that characterizes systems consolidation has been observed with some types of explicit memories – such as aversive memories, but not with other types – such as spatial memories, that tend to display a non-graded RA (Sutherland et al., 2010; Winocur et al., 2013), which also represents a challenge for SMSC core concepts. Actually, even in human studies there is some debate about what “episodic” really mean, with permanent HPC dependency being observed mainly in episodic memories of the autobiographical type (for a discussion, see Teyler and Rudy, 2007; Rudy, 2009).

Interestingly, both SMSC and MTT drank to some extent from the same HIT (Teyler and DiScenna, 1986; Teyler and Rudy, 2007), a very consistent early attempt to conciliate psychobiological data with neuroanatomy-of-the-day (Squire et al., 1984) plus some mathematical modeling of neural networks from the beginning of the 1970s (Marr, 1971) in order to explain the role of HPC in memory storage. HIT allowed, among other advances, the maturation of decisive concepts such as *pattern completion* and *pattern separation* (Teyler and Rudy, 2007). Another strong influence came from the seminal work of O’Keefe and Nadel (1978) that proposed the role of *Hippocampus as a Cognitive Map*, and the so called Complementary Learning Systems framework, which suggested a protective role for the HPC/NCTX interplay working to prevent catastrophic interference among similar patterns (Marr, 1971; McClelland et al., 1995; O’Reilly et al., 2014). Actually, despite invisible for many, it can be said that no modern theory of memory in cognitive psychology would exist today free from the influence at least two conceptual paradigms, the *information*-*processing* approach (e.g., the computer metaphor for the brain) – to this day, by far the most influential of the two (but perhaps on the negative side) – and the *connectionist* approach (e.g., parallelism, emergentism, neural networks, etc – see chapter 1 in Galotti, 2018), still scarcely explored.

## Systems Consolidation Dynamics: Exceptions and Alternative Models

Contextual fear conditioning and, in special, spatial learning, are among the behavioral tasks that produce more contradictory results in relation to the systems consolidation framework – i.e., they frequently produce *flat or non-graded temporal gradients* (Sutherland et al., 2008, 2010; Broadbent and Clark, 2013; Winocur et al., 2013), i.e., memories that never exhibiting independence from the HPC when retrieved. Sutherland et al. (2010) have even proposed an alternative model that would complement MTT and explain away diverging findings – the so-called DRT, according to which, instead of the “gradual and lengthy memory reorganization” of one single mnemonic entity, what happens is the rapid establishment of a dual-trace in both brain regions, with a stronger representation in the HPC, and a weaker one in the cortex. This would explain memory retrieval without an active HPC, since an extra-hippocampal trace, despite weaker, could yet be expressed in some situations. This interesting *ad hoc* hypothesis reintroduces an assumption already present – but frequently understated – in the SMSC (Squire and Alvarez, 1995), that is fully consistent with several other findings from our lab over the years (Jerusalinsky et al., 1994; Sierra et al., 2017 – see below): *cortical areas must be recruited simultaneously* with the hippocampal system during acquisition/learning in order to, later, support the temporally graded “changing of the guards” between the HPC and the NCTX, i.e., the suggested *dual trace* seems to exist at last.

One interesting conceptual suggestion originally proposed by MTT was that each time retrieval takes place, that trace would be automatically re-encoded (i.e., “re-indexed”) in the HPC, meaning that the older the memory, the more “copies” of its index would be available and the easier would be to retrieve then, in thesis (Nadel and Moscovitch, 1997, 1998). This idea was devised to explain, for instance, the robustness of some old memories, or for, say, memory of items reinforced by repetition or “rehearsal.” This interesting theoretical prediction, consistent with the best supporting ideas advocated by HIT (Teyler and Rudy, 2007), would be useful to account for several findings in the field of *memory reconsolidation* (Lewis, 1979; Nader et al., 2000a,b; Anokhin et al., 2002; Walker et al., 2003; Duvarci and Nader, 2004; Lee et al., 2006; Rose and Rankin, 2006; Hupbach et al., 2008; Bustos et al., 2009, 2010; Nader and Hardt, 2009; Nader and Einarsson, 2010; Hardt et al., 2010; Lee, 2010; Alberini, 2011; Haubrich and Nader, 2018) – indeed, an updated trace might even end up being expressed just as one of those index copies, slightly modified.

However, to our notice, notwithstanding the expected technical difficulties, this promising idea was never put to real test. The multiple copies scenario could, for instance, be contrasted with opposite theoretical models such as the CTT, also inspired by HIT (Yassa and Reagh, 2013), in which the HPC, through a memory reconstruction process called *recontextualization*, compensate for the deleterious effects of the competition among partially overlapping traces of aging memories, strengthening memories by semantization at the expense of contextual details.

In 2010, Winocur et al. (2010) advanced a extensively revised version of MTT – dubbed as the TTT – to incorporate the now widely accepted idea that the corticalized single episode trace is *not a mere duplicate* of the previous hippocampal version, but a *transformed* record with quite different characteristics. The transformation hypothesis differs from SMSC in that (1) it accepts the permanent HPC-dependency of detailed/autobiographical episodic, contextually bound memories, (2) the “hippocampal memory” supports the corticalization that produce a contextually poor, gist-like (“schematic“) engram, and (3) that HPC-related precise memories dynamically interact/compete for dominance with cortex-related generalized traces depending on the boundary conditions in the retrieval session (Winocur et al., 2010; Sekeres et al., 2018). The first two points were inherited from MTT, but the last one is new, and incorporates the very recent paradigm that emphasizes the parallels between HPC/precision and corticalization/generalization, i.e., the supposed connection between the neuroanatomical and the psychological/qualitative points of view.

## Two Complementary Approaches to Systems Consolidation

Then, coinciding with the gradual HPC disengagement in contextual fear memory expression, a number of studies have found that animals are good at discriminating between the original training context and a novel context shortly after training, whereas some weeks later they show equally robust conditioned responding to both contexts, an example of loss of contextual precision (Biedenkapp and Rudy, 2007; Wiltgen and Silva, 2007; Winocur et al., 2007). The reduced HPC engagement and the increased generalization in the cognitive domain may be more than a simple coincidence, and has been suggested to reflect a specific role for the HPC in mediating detailed, discriminatory memory expression (Wiltgen et al., 2010). In this line, progressive corticalization comes at the price of having most of the details of the original experience stripped off, attaining a more generalized nature. In the limit, we may suppose this is the first step in building *schemas –* a class of fast-response cortical psychological construct which goes far beyond a mere case of generalization of information, once they act by structuring both the information gathering and their use (Ghosh and Gilboa, 2014). The transition from memory discriminative precision to generalization may be used as a measurable psychological correlate of the temporally graded neuroanatomical involvement in systems consolidation. Notwithstanding its utility as an additional tool to study the phenomenon, attention must be paid in every experimental design to avoid false positives due to the fact that there are other ways to produce the generalization of any learned information: a series of time-independent generalization protocols such as sexual hormone levels, presynaptic GABA-B inhibition or the so-called cue-induced generalization do not correlate with systems consolidation and might deserve additional control groups in some experimental designs (see Jasnow et al., 2017).

In the following sections, we will review some results from our lab that, over the years, have raised some interesting questions possibly relevant for a discussion on the nature of the engram. After replicating the phenomenon from the *neuroanatomical point of view* in two different experimental setups, finding a similar time frame between 4 and 6 weeks – despite specific differences between the protocols – we managed to *accelerate* the transition of the retrieval control from HPC to NCTX (in this case, the anterior cingular cortex) simply by increasing the amount of learning opportunities between training and remote test sessions. We then explored other, different ways to modify the time course of systems consolidation, such as reactivating the main aversive memory. In between, we investigated the need for the lately engaged neocortical area to be actively involved already during the acquisition of the behavioral task.

## Close Encounters With Systems Consolidation

In the beginning of the 1990s, a time in which the Standard Model was still being formulated (McDonald and White, 1993; McClelland et al., 1995; Squire and Alvarez, 1995) and the phenomenon of systems consolidation wasn’t even named (Dudai, 1996), when studying the role of glutamatergic and GABAergic receptors in memory formation and expression, we found that the AMPA competitive antagonist CNQX was amnestic when infused into the HPC (and amygdala – in a joint, bilateral infusion) at 1, 6, 13, 20 but not 31 days after training (Bianchin et al., 1993; Izquierdo et al., 1993a,b; Quillfeldt et al., 1996), while the same blocking effect tend to last more when injected into the ERC, effective at 1, 26, 31 but not 60 days after training (Ferreira et al., 1992a,b; Jerusalinsky et al., 1992; Quillfeldt et al., 1994, 1996). Thus, HPC and ERC appear to have naturally “switched” their roles in memory retrieval somewhere between the 20th and the 31st post-acquisition day, at least for this specific aversive task (step-down inhibitory avoidance). The fact that cortical areas were displaying sensitivity to CNQX also *before* the HPC disengagement may be due to the drug of choice and the essential nature of the glutamatergic transmission in the CA1 area (a more detailed discussion appears in the end of the section entitled “New learnings before the remote test accelerate systems consolidation”).

This 2–4 weeks’ interval for the disengagement of the HPC is consistent with several studies involving rodents in contextual fear learning (Kim and Fanselow, 1992; Maren et al., 1997; Shimizu et al., 2000; Wiltgen et al., 2010; Beeman et al., 2013). There are, however some important contrary findings, reporting “flat” temporal gradients (Sutherland et al., 2008; Broadbent and Clark, 2013), but besides relevant differences in experimental protocols, some of these inconsistencies may be due to the fact that most of them have employed chemical lesions, which differ from our use of pharmacological reversible blockings, both in its extent and the possible outcomes (Sutherland et al., 2010; Goshen et al., 2011; Doron and Goshen, 2017). Anyway, once even humans display different durations of RA caused by comparable hippocampal lesions (Spiers et al., 2001a,b; Cipolotti and Bird, 2006), a similar variability in experimental animals is more than expected, specially among different strains, even local substrains of experimental animals.

**FIGURE 1:**
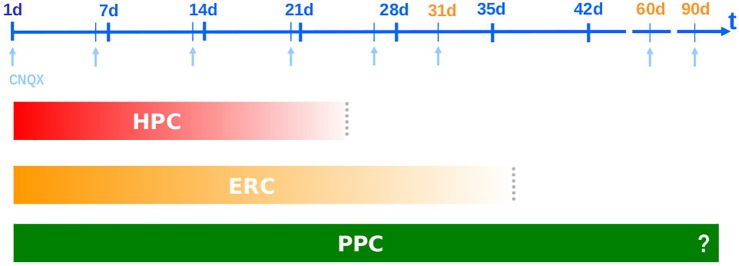
Systems Consolidation: CNQX blocks the performance in the Step-Down Inhibitory Avoidance task when infused into three phylogenetically distinct brain areas – dorsal hippocampus (HPC), entorhinal cortex (ENT) and posterior parietal cortex area 2 (PPC) – resulting in different temporal frameworks: neocortical area remains in charge of memory retrieval after hippocampal and entorhinal disengagement (Quillfeldt et al., 1994, 1996; Izquierdo et al., 1997).

On the other hand, in those old studies it was remarkable to notice how cortical areas use to display a longer involvement than the HPC: thus, while the ERC was sensitive to CNQX amnestic effect at 1 (Jerusalinsky et al., 1992; Izquierdo et al., 1993a), 26 (Quillfeldt et al., 1994) and 31 days after training days (Quillfeldt et al., 1996), the PPC remained responsive after 60, and even up to 90 days post-training (unpublished results). The “stepwise” or gradual “deactivation” of the involvement of these brain structures takes place in full agreement with their neuroanatomical hodology (Marr, 1971; McNaughton and Nadel, 1990; Buzsáki, 1996; Fuster, 1997) and, consistently, with their phylogeny (Sherry and Schacter, 1987; Lavenex and Amaral, 2000; Treves, 2009; Thome et al., 2017). Figure 1 summarizes these first findings.

There is a hierarchical organization in this time frame. Just as during learning/acquisition the sensory information flows first from multiple polymodal neocortical areas toward the paleocortex (entorhinal), and from there to the fast and iterated local circuits of the archicortex (HPC), now the processed information projects back to the associative NCTX through a paleocortical relay looking for a long-lasting storage site, closing a hierarchical loop (Teyler and DiScenna, 1986; McClelland et al., 1995; Lavenex and Amaral, 2000). This is why HPC, despite evolutionarily older, is considered the highest level of associative integration in the mammalian brain (McNaughton and Nadel, 1990) and the CNQX blockage experiments somehow unveiled the same timeline of the above hierarchical loop (Lavenex and Amaral, 2000). In this case, the representative of the associative NCTX was the posterior parietal area whose long-lasting responsiveness is in accordance with a putative role as the final residence for the engram.

## Temporal Framework for the Hippocampus Involvement: Rigid or Flexible?

Recently, we decided to revisit those original findings in our lab, asking *why* the HPC would need this particular time window of (then) 3–4 weeks to disengage itself from the retrieval process, originally in terms of AMPAR-mediated mechanisms, but other systems could be approached, such as the A-GABAergic one (Haubrich et al., 2016). We began by trying to replicate the above findings, but modifying three things, the drug (muscimol instead of CNQX), the aversive task (CFC) and the cortical target area: the ACC integrates the mPFC, a region that has been suggested to be of primary importance to support remote, but not recent memories (Frankland et al., 2004a,b; Teixeira et al., 2006; Ding et al., 2008; Insel and Takehara-Nishiuchi, 2013).

As consequence of these new experimental conditions, we detected a slightly longer time frame for the interplay between HPC and ACC – 4–6 weeks: HPC infusion of muscimol was amnestic in CFC-trained animals when tested at 1, 20 or 35, but not 45 days after training, while the same drug infused into the ACC produced the exact opposite scenario, being effective only at 45, but not 1, 20, or 35 days after training (Haubrich et al., 2016). This temporally graded phenomenon, despite slightly longer, still is compatible with previous findings, and represents a clear-cut instance of the systems consolidation phenomenon, despite not favoring any of the two main theories in dispute, the standard model or the MTT (Nadel et al., 2007).

## The Synaptic Occupancy/Reset Theory

Our main hypothesis was that **the duration of systems consolidation would be defined by the extent of use of the available synapses in the HPC**. Its testability, despite virtually impossible two decades ago, is becoming increasingly feasible now with the availability of high-tech tools such as opto/chemogenetics, multielectrode arrays and two-photon microscopy, despite still lacking the necessary spatial and temporal resolution (see the last section, “Testing the Theory,” bellow). Of course there may be alternative explanations for our findings, but synaptic availability represents a simple, straightforward and reasonable putative model, enough to prove being valuable to explore in more depth.

Motivated, as others before, by the Hippocampal Indexing Theory (Teyler and DiScenna, 1986; Teyler and Rudy, 2007), that, as mentioned above, was a quite successful theoretical approach absorbed in different degrees by most theoretical appraisals of memory systems consolidation (SMSC, MTT, DRT, TTT, CTT, etc.), we propose that:

(1) considering that **learning a new task equals to “connecting” a set of sensory inputs (S) to a set of motor outputs (M)**, a form of higher order “pavlovian” link, that will be summoned into action in some coordinated way during retrieval via the establishment of an *intermediate plexus* (IP) *of neural pathways* that produce the correct/learned response;
(2) considering that **those pathways would embody** (a) the spatial representation of the learned context (if learned), (b) the record of important items and subjects present, and (c) a set of efficient motor choreographies to be summoned in order to deal with what is being perceived in that moment, all these components will, at the end, assist a decision taking based on matching/non-matching between the present sensory inputs and the stored memory (Fernández et al., 2016; Agustina López et al., 2016; Krawczyk et al., 2017);
(3) considering that the HPC is such a **small brain region** in terms of number of neurons (thus, number of synapses available at each moment) – particularly in the rat (Braitenberg and Schütz, 1983; McNaughton and Nadel, 1990; Treves and Rolls, 1994; Rolls et al., 1998; Rolls and Kesner, 2006; Treves, 2009; Rolls, 2017), and
(4) considering also the **ever-growing amount of data to be continuously encoded** by any normal animal, even for experimental ones.

We hypothesize **when submitted to a rich, successive series on new learning situations, the hippocampal system would easily reach maximum occupancy and might need some special maintenance**: the simplest way to do this [considering first a fixed (or restrict) set of available synapses] would be to *free* synapses previously engaged in some other representational index to become again available to hold the new memories – a kind of **synaptic reset**. In this *occupancy-reset* scenario, hippocampal synapses *might endure physical erasure* in at least two basic situations: (a) *on demand*, when the number of available, unoccupied synapses reaches a minimum, not enough to hold a new engram/trace, reset would “make space” to continue the storage process, or (b) *automatically, on a regular basis*, in the case of an “uneventful, tedious life” – typical of experimental animals that usually live for just one lifetime experience, a quite unrealistic, non-ecological situation, as Ulrich Neisser has alerted before (Neisser and Winograd, 2006) – a portion of this synaptic population would be automatically reset from time to time, a natural turnover, which could explain the timeframe of the “natural” systems consolidation observed in different experiments.

Of course this is just a first sketch, with the minimum components necessary to accommodate the experiments described in the sequence. A more detailed proposition appears in the last sections of the paper. To this point, among several assumptions, there is one that is in full accordance with HIT: the HPC will not encode the full trace of an experience inside its borders, holding just a *map* to the true location of the engram in the much more extense neocortical associative areas. The first premise above is also an epistemological commitment with the psycho-physical identity principle, a position in line with philosophical materialism, realism and systemism (Bunge, 2010), that receive different names in the scientific context, such as the “principle of functional-neural isomorphism” (Sekeres et al., 2018), when referring, for instance, to things such as the interplay between psychological phenomena and their neural representations.

## *New Learnings* Before the Remote Test *Accelerate* Systems Consolidation

With a well-defined systems consolidation experimental setup at hand – and if the *synaptic occupancy/reset* hypothesis is correct – we might next ask *why* does this phenomenon has this specific duration of 4–6 weeks (a period that encompasses both studies), at least for rats and in these aversive tasks. One logical possibility, derived from the finiteness of the HPC itself, would be to consider, for starts, that the number of synapses available to encode new memories is *finite and fixed/restrict*. Since these synapses should be “ready” for plasticity events, maintenance activities must be performed regularly, and we suggest that there may exist a regularly scheduled automatic “reset” of these synapses. This would naturally destroy previously used index mappings of cortical engrams, meaning that those memories would be physically deleted. Although there can be reasons to despise the omnipresent computer metaphor in the neurosciences, it is hard to resist an analogy to describe this maintenance-reset-induced-amnesia: the deletion of the FAT table in a computer’s hard drive does not remove the bits of memory actually spread/intermingled all over the disk, but renders that memory virtually unrecoverable due to the loss of tracking information. We hypothesize that a similar process would be taking place in the CA1 HPC pyramidal neurons, responsible for establishing the index of each memory trace and keep track of their spread parts. So the first prediction of the s*ynaptic occupancy/reset theory* (SORT) is that **forgetting is just a natural consequence of this natural maintenance mechanism** (at least the passive component). The average 4–6 weeks period would represent the automatic (predetermined or scheduled) reset/erasure, once the animal is not being trained in any other task and would not be “using” those available synapses.

Next we ask: what if we interpose a series of novel learning tasks between the training and the remote test sessions? Hypothetically, this would forcefully “increment the cognitive life” of this experimental animal, and more synapses should be in demand: if the minimum limit of available synapses happens to be reached during a series of intense cognitive experiments – a reasonable supposition considering the small dimensions of the HPC – this would trigger the reset system *before* the regularly scheduled moment in order to release more, fresh synapses to build new memories. Thus the second prediction would be that **systems consolidation would endure an acceleration**, with the *switching point* that disengages the HPC and summons the neocortical areas moving to a time point *before* the completion of the regular *interval of* 35–45 days after training. The learned memory would become independent from the HPC, and dependent on a cortical areas such as the anterior cingular cortex at an earlier time point. This neuroanatomical displacement could be verified employing muscimol to check for the involvement of each brain structure at an earlier time point, say, 20 days after training.

**FIGURE 2:**
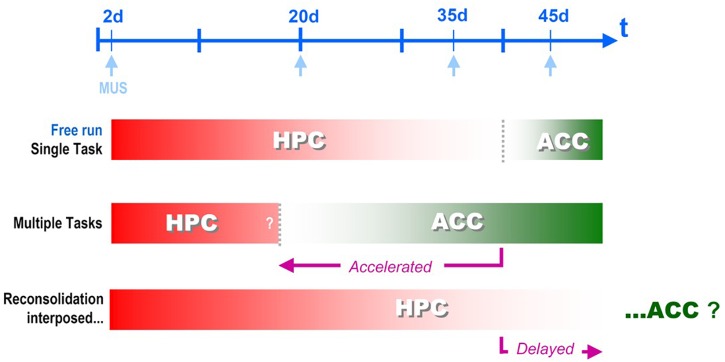
Systems Consolidation temporal framework, i.e., the time for the corticalization onset after hippocampal disengagement, can be flexibilized by different behavioral interventions interposed between the training and the remote test sessions for Contextual Fear Conditioning memory retrieval: Muscimol displays different windows of effectivity when infused either into the dorsal hippocampus (HPC) or the anterior cingular cortex (ACC) depending the nature of the interposed sessions - new learning or reactivation/reconsolidation (De Oliveira Alvares et al., 2012, 2013; Haubrich et al., 2016).

**FIGURE 3:**
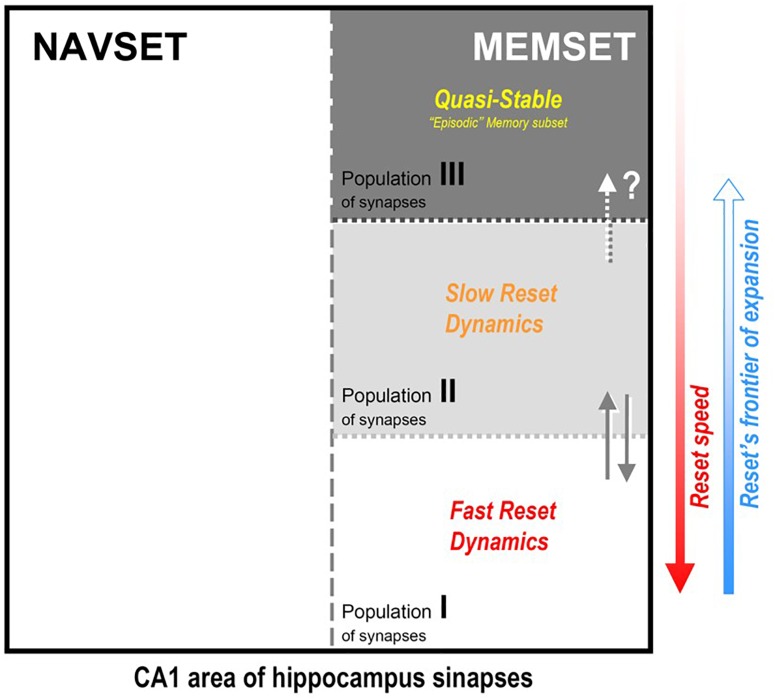
Hipothetical internal organization of CA1 hippocampal area according to the synaptic occupancy/reset hypothesis (SORT, as described in the text): all active synapses would be divided in two functional (not spatial) subsets, the navigational (NAVSET) and the memory indexing (MEMSET) subsets, that might overlap to any extent (multitasking neurons). Memory-recording synapses, on their turn, might be divided in subpopulations with different degrees (speeds) of “resetability”, here displaying three of them, those with faster and slower reset dynamics, and the quasi-stable one. Since MEMSET would consist of a finite, relatively constant number of synapses, the more memories exist to demand encoding/indexing, the more free, “fresh” synapses are needed, thus forcing the reset process to progressively encompass more stable synapses populations. Three population is an arbitrary division just to prove concept, once resetability might even be a property that varies continuously among the whole ensemble of MEMSET synapses.

**FIGURE 4:**
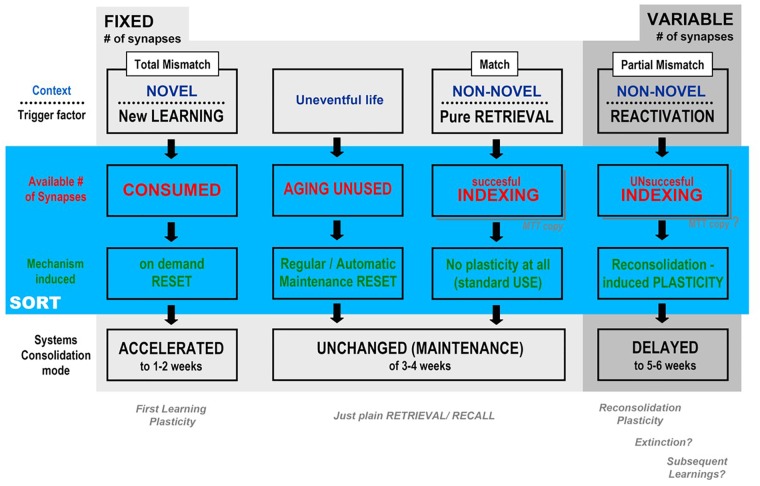
Synthesis of the possible outcomes, according to the degree of contextual mismatch presented by the tasks interposed between the training and remote test sessions of the main, aversive task (e.g., CFC), according to SORT (described in the text): Departing from a fixed, finite number of CA1 synapses in MEMSET, multiple sessions of novel context presentations (new learnings) would involve more and more new information, thus demanding the reset of previously used synapses in order to “make room” for new memories, *before* the natural, maintenance reset cycle, which *accelerates* systems consolidation temporal framework. When context is recognizable (partial mismatch only), different plasticity mechanisms would be triggered that *adds* extra synapses to MEMSET in order to cover the encoding needs without engaging in “reset on demand”, which would explain the observed delay in systems consolidation (see Figure 2).

The result was exactly what was expected! Muscimol infusions showed us that CFC memory became independent from the HPC before the regularly scheduled time (Haubrich et al., 2016 – see Figure 3E), and was now relying upon the ACC area (*ibidem*, Figure 4E), i.e., systems consolidation was *accelerated* by multiple learning experiences, consistently with the *occupancy/reset theory.* See Figure 2, ahead.

This underlying hypothesis was barely sketched in the original paper (Haubrich et al., 2016): “We hypothesized that the encoding of multiple memories would result in an accelerated HPC-to-cortex information transfer in order to preserve hippocampal function of encoding new information and avoid its overload” (…) “It may be that such rapid reorganization occurs in order to preserve hippocampal storage capacity, allowing the HPC to continuously process new information, given that its physical storage is likely limited. This may also reduce *interference* with previously established memories.” The mention to “interference” was another echo of the precursor ideas of Marr and McLelland’s pioneer propositions (Marr, 1971; McClelland et al., 1995).

In support of these findings it was shown that multiple learning experiences may induce changes both in dendritic spine complexity and c-fos expression in the ACC at delays that resemble those of our remote memories (Wartman and Holahan, 2013, 2014). Most important – and a strong support for the main tenet of the indexing theory – a central role for the HPC was demonstrated in the active induction of neocortical plasticity related to memory processing, i.e., the accelerated HPC-to-ACC memory reorganization may be under control of the HPC itself (as suggested by Sutherland et al., 2010). There might exist alternative explanations for these results as, for instance, new learning inducing competition for hippocampal storage room as a side effect of the memory allocation process upon the excitatory, principal neurons of neocortical networks (Han et al., 2007; Josselyn and Frankland, 2018). For now, however, our favorite candidate mechanism for the reset mechanism might rest in processes such as neurogenesis (see, e.g., Besnard and Sahay, 2016), already shown to be induced by novel learning (Gould et al., 1999a,b; Kitamura et al., 2009). Of course, a lot more remains to be investigated.

Comparing the two sets of experiments separated by 20 years, the main difference between them was in the *duration of the observed drug effect* (compare Figure 1 and 2) probably due to the chosen neurochemical target. In the previously mentioned works, we have prioritized AMPAR for the pre-test blocking of retrieval, while muscimol was used only for the post-training infusions in order to evince consolidation effects (Quillfeldt et al., 1996; Izquierdo et al., 1997). Due to the existence of a similar circuitry arrangement both in the HPC and the NCTX, in which GABAergic interneurons control pyramidal glutamatergic cells through feedback and feedforward inhibition in simple, yet reliable local circuits (Pitler and Alger, 1992; Bull and Whittington, 2007; Spruston, 2008; Tremblay et al., 2016), the infusion of the GABA agonist muscimol was expected to reversibly suppress local activity (either in the CA1 area of the HPC or the NCTX), more or less the same way the AMPAR antagonist CNQX would do: the first, by stimulating GABAergic interneurons, and the last, by directly blocking glutamatergic principal neurons. However, we should consider the possibility that plasticity might have modified the level of response of these systems in different ways. Thus – and particularly in the HPC – while the responsivity of (at least some) interneurons could be reduced to near zero without drastic consequences, the same might not be possible for the principal neurons, once they happen to be the only available carrier pathway for the output of hippocampal processing.

In other words, GABAergic neurons would be freer to vary their connectivity than the principal, glutamatergic cells, and this is an decisive point since *we are studying the pharmacology of this local neural circuit, not individual, isolated neurons*. This aspect might account for the fact that CNQX *was amnestic from day* one both in the HPC and the cortical areas, ending its effect in the ERC (but not in the PPC) at the moment the structure was apparently “released from duty” (Quillfeldt et al., 1996), while in the 2016 experiment, Muscimol was effective in ACC only after the HPC ceased its engagement with memory retrieval (Haubrich et al., 2016). To some extent, the more recent experiment sounds more convincing and representative of systems consolidation, but whenever the unexpected happens, there is opportunity for a deeper peep into the processes under scrutiny: thus, these two similar, yet not identical ways to observe systems consolidation for an aversive task teaches us two additional things: (1) at least in terms of glutamatergic transmission, *cortical areas appear to be necessary from the very beginning*, right after acquisition, even if this is not apparent in every chosen experimental design, and (2) GABAergic modulation might be the locus of the central plastic events behind the transference of function observed in systems consolidation, that would be the reason why its manipulation results in a clear-cut systems consolidation time frame in both brain structures. Observation 1, for instance, have received additional support from at least two previous works of us, for instance, in Jerusalinsky et al. (1994) and, more recently, we studied a remote memory blocked by pre-training infusion of muscimol into the ACC, and managed to use reactivation/reconsolidation to rescue the supposedly lost trace and also restore the normal course of a disrupted systems consolidation – a putative case of “systems re-consolidation” (Sierra et al., 2017). The need for the presence of neocortical areas from the very beginning – despite only mobilized later in the systems consolidation process, is another exciting subject that, however, will not be further discussed here.

## *Reactivation Sessions* Before the Remote Test *Delay* Systems Consolidation

Under specific protocols of re-exposure to the original training context, *reactivation may* take place *during* memory retrieval and a memory that was previously acquired and already fully consolidated (in a synaptic consolidation process) would be *relabilized, becoming* again sensitive to modification or even disruption. This allows for the integration of new information (update) and the process concludes with the *reconsolidation* of the former trace into a modified engram (Nader et al., 2000a,b; De Oliveira Alvares et al., 2008a,b; Bustos et al., 2009, 2010; Lee, 2010; Alberini, 2011). In our lab, we have been studying reconsolidation for some time, and have found, for instance, that, during a reactivation session, the concomitant presence either of a distractor (Crestani et al., 2015) or an appetitive stimulus (Haubrich et al., 2015), was able to promote a long-lasting reduction of freezing response, i.e., effectively modify the emotional valence of the originally learned tasks (CFC) to a less aversive level. In those two studies, the effects were abolished either by systemic nimodipine, or intra-hippocampal infusion of ifenprodil, which is consistent with a reconsolidation mechanism: LVGCCs, and, specifically, GluN2B-containing NMDARs appear to be common plastic components recruited in the HPC by these two different cognitive situations, once its blockage interfered with memory reconsolidation. Using reactivation/reconsolidation we have also managed to incorporate an endogenous state-dependency into previously consolidated memories (Sierra et al., 2013) and use reconsolidation to promote the consolidation of a concomitant weak learning through a synaptic tagging and capture mechanism (Cassini et al., 2013).

But memory “flexibilizing” protocols may also be employed to interfere with higher order cognitive phenomena, such as systems consolidation, in which multiple brain areas are recruited in a complex spatio-temporal choreography of engram-allocation. Two examples from our lab have managed to successfully interfere with the temporal framework of systems consolidation by inserting short *reactivation sessions* between training and the remote test (De Oliveira Alvares et al., 2012, 2013). In this experimental setup, despite checking only for one brain structure (the HPC), systems consolidation was “measured” by the psychological, qualitative modification in the ability to discriminate between original and novel contexts as advanced, e.g., by Wiltgen and Silva (2007). In other words, if memory has precision, the muscimol infused into the HPC must suppress that precise response and the animal confound the conditioning context with the novel context. At later periods, generalization (corticalization) would have take over the process and the animal would naturally not be able to discriminate between the contexts (and the HPC would become insensitive to pharmacological blockage).

And then systems consolidation was once again replicated! This time measuring the precision/generalization psychological binomium. In the training-test interval of 2 days, animals were able to discriminate well between known and novel contexts (i.e., display precision), and muscimol suppressed this capacity when infused before test into the CA1 region of the HPC (i.e., display HPC-dependency). The experimental group tested after 28 days – an interval in which the HPC was not expected to be responsible for retrieval anymore – animals did not discriminate between the contexts (i.e., they exhibit memory generalization) and muscimol did not produce any response (i.e., we detect independency from the HPC). Despite not studying any cortical target in this case, the results were a clear reproduction of half the systems consolidation viewed from the HPC perspective (De Oliveira Alvares et al., 2012 – see Figure 1), and consistent with previous contextual fear generalization studies (Biedenkapp and Rudy, 2007; Wiltgen and Silva, 2007; Winocur et al., 2007),

Next we asked what would happen *if we interpose reactivation sessions* between the training and the remote test sessions. Subjects trained in CFC were reexposed to the original training context in the absence of the unconditioned stimulus (footshock) for three sessions of just 90 s each, once a week. After these reactivations, the control group (vehicle-injected) became again able to discriminate between the novel and the conditioning contexts, notwithstanding the long interval that normally would have lead to the corticalization/generalization of the memory trace. When muscimol was infused in the CA1, however, the ability to discriminate was gone, showing that HPC was again in charge of retrieval of this otherwise remote memory (De Oliveira Alvares et al., 2012 – see Figure 2).

What happened here was somehow unexpected: the HPC-dependent, precision-prone period was literally *enlarged*, i.e., the systems consolidation temporal framework was *delayed*, the exact opposite of what the new learnings have produced. The same delay was observed in another experimental setup in our lab with the insertion of *only one short reactivation session* (De Oliveira Alvares et al., 2013 – see Figure 3). In this second discriminative experiment, the re-exposure session was proven to consist of a real reconsolidation process of the original memory trace, since the delaying effect was suppressed by nimodipine injected i.p. before the reactivation session.

In sum, compared to new learnings, reconsolidation has produced an *opposite* effect upon the temporal framework of systems consolidation. Notwithstanding some similarities, such as protein synthesis dependency, there is an increasing list of intrinsic differences between first-time consolidation and reconsolidation, involving different membrane-bound receptors and channels, membrane insertion of ion channels, enzymatic degradation cascades, early genes, etc. (Haubrich and Nader, 2018). These differences could explain several different outcomes in different scenarios, thus observing a delay instead of an acceleration should not come as a surprise, despite not having been anticipated by the present version of the *occupancy/reset hypothesis* straightly *based on the assumption of a fixed number* of available synapses. Actually, (a) since reconsolidation should necessarily involve some degree of synaptic reorganization in order to update the original memory trace, and (b) since synaptogenesis is not an uncommon event in the hippocampal area, even out of the context of developmental critical period, we may hypothesize that the (different) kind of plasticity elicited by reconsolidation may result in an equally different outcome for instance, a direct increase of the total number of available synapses in the immediate neighborhood of the “reconsolidated“ cells, at least within certain limits (there is no room for an indefinite increase of this number). In other words, there could be a second, alternative mode of operation of the set of available synapses other than that controlled by the occupancy/reset putative mechanism, now based on the complementary assumption of a variable number of available synapses.

Of course, this is highly speculative, but at least, is testable. One consequence of the local variation in the number of available synapses without the need to recruit by reset upon a fixed set would look as an expansion of the CA1 area involvement and result in a systems consolidation delay that fits what was observed in the reactivation/reconsolidation experiment (De Oliveira Alvares et al., 2012, 2013). Figure 2 summarizes the two opposite findings that resulted either in acceleration, or delay of the systems consolidation process:

The hippocampal indexing theory suggests that this operation should tackle upon the same index of the original memory, maybe adding some extra connections here/removing others there, an operation that could or could not demand more available synapses to take place. And, as we discussed briefly above, MTT has the interesting proposition that each time a memory is retrieved, a new index would be created as a partial copy of the original trace plus some additional features integrated as an “update,” a way to explain the resilience on older memories: but if reconsolidation creates a new index, it would demand more synapses and should contribute to move the ensemble of plastic, available synapses closer to the limit of occupancy, which would result in a *reset on demand* and the acceleration, not the delay of the temporal framework. Since this has not happened, something else should be going on. We can improve our model by adding another feature to it: the capacity to create new synapses, at least within certain limits (once HPC CA1 area is still such a small structure). That would be the first thought of most researchers since we use to feel comfortable with the idea that there is a “free capacity” to “produce more” (synapses, cells, etc.) and intuitively (and acritically) we comply to this comfortable position. But this may not be true.

Thus, having begun with a restricted model in which new memories must be recorded making use of a finite number of available synapses in order to survive, we have to warrant available room for the creation of *more* substrate for engram plasticity: in the above situation, if the number of synapses were *not fixed*, but variable, an increment would easily explain the delay. Maybe that is what takes place in the specific case of memory reactivation by partial mismatch of contextual cues (Fernández et al., 2016; Krawczyk et al., 2017), differing for the new learning situation, where a *total mismatch* is verified and lead to memory formation (upon a fixed set of plastic synapses). Again, be it real or not, this is a testable complementary hypothesis. We can think of it as a “toy model” designed to tie some loosen ideas and experiments with new, putative (hopefully reasonable) integrative conceptual ideas. Good theories should prioritize simplicity whenever possible, and ensure at least three things: *explainability* (have no contradictory findings), *testability* and *predictability* (Bunge, 1967, 1985). Any one of these properties is of paramount importance, and the absence of one of them will strongly limit any proposition. However, people tend to focus more on the first two properties, neglecting predictability – maybe the most important of the three. We will discuss a more complete version of the model in the next session, but before, let’s bring some closing remarks on the experiments here discussed.

These last two experiments raise an important question, and even a possible objection to what we have found in the acceleration-by-new-learning experiment (Haubrich et al., 2016): couldn’t it be the case that what was actually taking place was some instance of reactivation, not the mere accumulation of information that would demand more “synaptic room”? This would also make things complicate for the synaptic occupancy/reset theory, and the possibility was not directly tested in the original 2016 experiment. However, we can mention at least three reasons to reject this alternative explanation. First, both interposed tasks, despite intentionally chosen to be HPC-dependent, [a] does not involve re-exposure to the same context where CFC was learned, and [b] involve different (insufficient) exposure time, meaning that these additional tasks were unfit to reproduce the exact boundary conditions necessary to allow for a pure reconsolidation-dependent interference that could explain the observed change in the temporal course of the systems consolidation. Second, despite being far from attaining the exact boundary conditions, considering that those interposed tasks involved actual new learnings, the induced protein synthesis could provide, among its products, diffusible plasticity-related proteins (PRPs) that could be relayed to, somehow, produce interference via a tagging-like mechanism; however, the capture of PRPs might obligatorily take place in a short period, enough to interfere with late LTP maintenance, which would be very improbable after the several days that separate the original training and the interposed tasks. Finally, we must clarify that the Haubrich et al. (2016) paper was not our first attempt to study the consequences of multiple tasks interposed during a long-lasting training-test window, but it was the only one in which the protocol worked fine. In previous attempts, we have first tried to implement an intensive training protocol, with too many different tasks along the day, and even intercalating those tasks with long exposures to enriched environments: however, most of these animals resulted more stressed than “enlightened,” and the final results were inconclusive (data not published). Curiously, however, Lucas de Oliveira Alvares managed to implement an experimental protocol in which intentional stressful conditions (via aversive training intensity) was also able to accelerate systems consolidation (Pedraza et al., 2016), but the data we have did not support the idea that our multiple learning protocol caused any abnormal level of stress in order to compare both experiments. This last case of acceleration diverge from the interpretation we provided for new learning findings above, but this may be due to the more disruptive, maladaptive scenario induced, in which cells endure abnormal operation conditions (stress!) and may even suffer some degree of tissue destruction: to this point, our predictions concern mostly to healthy, non-pathological conditions, but those other conditions should receive further attention in future works.

## Hippocampus: Two Functional Sets, Three Dynamical Populations of Synapses (At Least)

Indexing theory was actually an elaborate attempt to explain episodic memory with the HPC at the center of the action. This small, yet fast-processing structure would be able to automatically capture contextual information, organize it in separate single episodes, and retrieve each one of these from a partial set of cues. These abilities are consistent with its highly and recursively interconnected nature that contrasts with that of neocortical circuitry – the supposed *final destination* of the memory trace – that, despite having much more neurons (thus, synapses) to make available, is too sparsely connected to support fast encoding and efficient retrieval (Rolls and Treves, 1998; Rolls et al., 1998; Rolls and Kesner, 2006; Teyler and Rudy, 2007; Treves, 2016; Rolls, 2017). In other words, the HPC solves the two main obstacles to the feasibility of episodic memories processing: the *associative connectivity* problem, that restrains NCTX, by allowing rapid *pattern completion*, and the *interference* problem between multiple, contextually similar episodic memories, by supporting *pattern separation* capacity (Teyler and Rudy, 2007; Moser et al., 2015).

Indexing theory is still one of the best possible general proposals for a hippocampal role in memory. Due to its finite dimensions – and consequent small number of neurons – particularly in the rat, this brain area will just be able to hold a small physical record consisting of a set of cortical “coordinates” or “pointers” – the *index* – and certainly never store the whole engram itself, not even temporarily (Squire et al., 1984; Treves and Rolls, 1994; Squire and Alvarez, 1995; Teyler and Rudy, 2007). This also harmonizes with the neuroanatomical-functional fact that this phylogenetically old area represents the *highest level of information integration* in the mammal brain: it receives converging polymodal sensory data from different cortical areas, first, the parahippocampal cortex, then, the entorhinal cortex; after the completion of the trisynaptic “data crunching” and the establishment of the index for that memorized experience – information flows back to widely dispersed associative areas of the NCTX, first via the entorhinal, and then, the perirhinal cortices (Lavenex and Amaral, 2000).

Figure 3 presents a more complete version of the *synaptic occupancy/reset theory*, integrating most of the relevant aspects it should contain, despite still sketchy and highly speculative to this point. From what we have already discussed, emerge some interesting hints and cues concerning the very nature of the engram, in the complex spatio-temporal framework of the systems consolidation process, whatever the engram may consist of.

Figure 3 illustrates the internal organization of CA1 hippocampal area according to the *synaptic occupancy/reset hypothesis* that we describe in more detail below:

(1) **Hippocampus CA1 area is the mandatory output way for any resulting pattern** of activity previously processed by the DG-CA3 subsystem, and its projection cells might connect indirectly (via a paleocortical relay) to neocortical target areas by encompassing and tying – through the available plastic synapses – a selected ensemble of projection pathways into the NCTX. **So the *synaptic occupancy/reset hypothesis* refer basically to CA1 principal cells and their ensemble of available synapses**, although the subset of synapses might well include other cells like interneurons, due to the intrinsically “circuital nature,” with local feedback/feedforward inhibition by the integration of different classes of interneurons with the principal neurons, as mentioned before;
(2) However, while the above suppositions aim to cover memory mechanisms in the HPC, that must not be the whole story for this fascinating brain region. If there is one function that is well established for the hippocampal formation and adjacent cortices is that of a ***Cognitive Map*** (O’Keefe and Nadel, 1978; Treves, 2009; Moser et al., 2015), a system that not only *organizes the representation* of the spatial context, but actually *creates* and imposes it to the surrounding space in which the animal moves/explores. This is used to anticipate needed adaptative maneuvers to be implemented, organizing real-time navigation with simultaneous well-structured (pattern-separated) capture of environment data. **This Navigational Set of Functions (NAVSET) must always be accounted for** in any theoretical proposition of any additional hippocampal function such as memory indexing. **The extra-navigational *Memory Set of Functions* (MEMSET)**, it is reasonable to admit, **might coexist with the navigational one in the same space, sharing many (if not all) neural cells**, as has been extensively suggested elsewhere (Marr, 1971; McClelland et al., 1995; Rolls and Kesner, 2006; Knierim, 2015; Moser et al., 2015; Lisman, 2017). This is a clear *dual function system*, and although both functions seem to be *inseparable*, at least they can be *distinguished* one from the other (thus, quantified) by employing the appropriate methodology (see Bunge, 1985, p. 28). Different sets of experimental approaches have actually been studying these distinct functional outcomes of the *very same* brain structure, the HPC, and due to its intrinsic complexity, even “distinguishing” different, parallel outcomes may present sometimes a spectacular challenge to science.
(3) MEMSET contains the principal cells and local interneurons, and their available ensemble of plastic synapses, all being capable of establishing some connections between themselves, but mostly with neocortical neurons (as described in item 1, above). **We propose that all these synapses are prone to be reset by the maintenance mechanism suggested before, but with different degrees of resistance to the *erasure* process.** So, there would exist at least three intermingled populations of synapses, two “dynamical” – easier to be reset, and one robust/resilient population of quasi-stable synapses that may explain long-lasting, detailed episodic memories. Three population types serve to illustrate the consequences of the hypothetic *differential resetability* (or erasure probability):
  **Type I** – **fast reset dynamical population**: for new and less relevant (“forgettable”) memories; holds most new memories, but mainly those that we easily forget, which is explained by the fact that in any reset/erasure session, these are the first to go;
  **Type II** – **slow reset dynamical population**: for new and mostly recent necessary/useful memories of all kinds; they last longer than population I, but do not hold forever, just enough to convey their needed information;
  **Type III** – **quasi-stable population**: despite being the most resistant to the reset procedure, they cannot be said to be “eternal” – quasi-stable is not the same as stable – and this is consistent with the fact that even episodic memories do recede and disappear with time, even in the extreme case of HSAM patients, despite their ultra-slow forgetting;
(4) The more demand for “fresh” synapses upon the MEMSET, the more cells/synapses would be recruited and submitted to the reset procedure, starting from population I and expanding into population II (and even to III, if necessary, at least partially); in other words, those **borders between populations are movable and there is a natural direction for frontier expansion**;
(5) The **division in three populations** with “three levels of resetability” **is, of course, purely arbitrary** and practical justification: three levels is the simpler non-binary classification, a simple, but effective way to describe a system that probably is much more complex; it may be the case that MEMSET consists, say, of 4, 5, 7, 12 – whatever – different populations, or it might even be the case that those cells/synapses obey a continuous distribution (which can be consistently classified back in a finite set of discrete bins like this to facilitate understanding).

The naming of these three populations of synapses came as an analogy to the classification of stellar population types in astrophysics (Trager et al., 2008): type I (*young*, metal-rich, orbiting inside the galactic bulge), type II (metal-poor, *old*, spread farther, in the galactic halo), and type III (metal-free, ultra-massive, *very old* or of hypothetical existence). Thus, type I synapse population include the “youngest,” continually recycled ones (by the reset), type II, the “older” ones, that takes more time to be reset, and type III are the “oldest,” reset-resistant ones, that might account for the phenomenon of HPC-dependency displayed by human episodic memories.

## Sketching Some Predictions

Besides providing a reasonable, fully HIT-compatible explanation of systems consolidation under three different frameworks – no intervention (free run), with new learnings, or with reconsolidation interposed between the training and the test sessions – SORT also imply some additional predictions:

• the model *elaborates upon one theoretical consideration discussed by the proponents of SMSC*, but not explored later by MTT or TTT: the putative connectivity changes not only can take place gradually, across weeks and months, but might also be “limited to expansion of the original axonal and dendritic fields or arborizations within these fields” (Squire and Alvarez, 1995): the functional link between HPC and NCTX is proposed as a limited, relatively fixed set of available plastic synapses that allows the selection of the correct subset of axonal projections from CA1 area to the respective cortical representations;
• the model *naturally explains both types of explicit/declarative memory dynamics – non-graded and temporally graded* – proposing the *very same underlying mechanism to explain both processes:* it explains why *episodic memories* would be prone to exhibit non-graded RA – their indexes would be supported by the so-called population III of quasi-stable, hard-to-reset synapses – and, at the same time, accommodates all the remaining temporally graded RA’s in the occupancy → reset paradigm; the old controversy between HPC-dependency and HPC-independent memories, that have lead to alternative models such as MTT and TTT, can now be explained by a simpler mechanism that also can be consistent with these previous theoretical propositions;
• the model *is compatible with MTT* in the sense that it supports the idea of extra, similar indexes being created by retrieval, and do not oppose – rather complement this hypothesis proposed to explain temporally graded RA of episodic memories (Nadel and Moscovitch, 1997); it actually goes one step further by providing a putative way of directly testing it (see next section);
• the model *does not explore (as SMSC have had* – Squire and Alvarez, 1995) *the idea that frequent or constant reactivation – or “replay” – of the trace during the delay period is necessary to effectively encode long-term memory, i.e., to complete the consolidation process*; this replay process is supposed to take place during certain phases of the sleep, such as the SWS (Rasch and Born, 2013; Sara, 2017), and involves, among other aspects, the sustained activity of neighbor areas such as the parahippocampal cortex (Schon et al., 2005) as well as muscarinic cholinergic modulation (Hasselmo, 2006), just to mention two important aspects that end up integrated in an interesting theoretical model of *rule learning* that involves, as an important component, the neuromodulatory regulation of presynaptic inhibition learning (Stern and Hasselmo, 2005; Hasselmo and Stern, 2018). Notwithstanding its importance, *replay* models exceed the scope of this paper, and will not be further discussed here: suffice is to say that this phenomenon is compatible with the model, and may even help to explain how available CA1 synapses are selected to create a memory index. However, the question of neuromodulatory regulation of presynaptic inhibition over local neural circuits has been studied in lab for many years: we also have delved into the cholinergic muscarinic modulation of memory, studying M4 action (Jerusalinsky et al., 1998; Ferreira et al., 2003; Sánchez et al., 2009), as well as endocannabinoid CB1 modulation (Quillfeldt and De Oliveira Alvares, 2015): both modulations act upon specific GABAergic interneurons in the CA1 area, and there are striking similarities in the way both modulations act. In the endocannabinoid study, we were able to show how the CA1 circuitry is affected in different phase of memory, operating as a “switching” mechanism between consolidation and retrieval processes, as well as between reconsolidation and extinction (Lee et al., 2006; Quillfeldt and De Oliveira Alvares, 2015);
• the model is fully compatible with the *memory reconsolidation phenomenon* (Lewis, 1979; Nader et al., 2000a,b; Anokhin et al., 2002; Walker et al., 2003; Duvarci and Nader, 2004; Lee et al., 2006; Rose and Rankin, 2006; Hupbach et al., 2008; Nader and Hardt, 2009; Bustos et al., 2009, 2010; Nader and Einarsson, 2010; Hardt et al., 2010; Lee, 2010; Alberini, 2011; Haubrich and Nader, 2018), for which there is an ever growing literature on putative mechanisms and specific “markers” that distinguish it from the consolidation of a first-learning situation; also, there are some studies on “systems reconsolidation” (Debiec et al., 2002; Wang et al., 2009; Einarsson et al., 2015; Sierra et al., 2017; Lopez et al., 2018);
• the model creates *a conceptual bridge between the two types of consolidation, synaptic and systemic*, generally treated as completely different phenomena (or phases of a wider process): by anchoring the formation of the hippocampal index simultaneously to a LTP-like process – at the synaptic level, and to the establishment of connections with efferents projecting to several sparsely distributed neocortical areas – at the systems level, the engram is formed at once, synaptically and systemically; these are not two independent phenomena anymore, but may be taken as stages of one only process;
• *forgetting* would be a straightforward consequence of this model, and if correct, might support proposals involving true erasure, specially of population I and II synapses (Rudy, 2008; Han et al., 2009; Clem and Huganir, 2010), an empirical situation that, otherwise, might be impossible to be conclusively proven, since we never could eliminate a purely negative hypothesis such as “the memory trace is not in the brain anymore”;
• the model is predictive also for *dementias*, such as Alzheimer’s disease, but on a first approach this would be quite trivial, because loosing neurons would automatically suppress available synapses in the HPC;
• more interesting would be to investigate its predictions in relation to pathologies involving stronger, sometimes self-reviving disruptive memories, such as those of PTSD (Ursano et al., 2010), whose mechanism might involve, for instance, the blocking/suppression of the reset mechanism – whatever it be – upon most cells/synapses, at least as part of the symptoms;
• similarly, but devoid of such devastating effects of PTSD, the very strong and detailed preservation of personal reminiscences as displayed by the HSAM individuals (LePort et al., 2012, 2017; Santangelo et al., 2018) can be consistently accommodated in this model, just by involving modifications in the reset system in population III synapses (notice that even HSAM do forget, i.e., loose details of highly details episodic memories, only extremely slowly);

## Putting All Pieces Together

Figure 4 is a self-explainable table of possible outcomes according to the different trigger factor, and covers most of the experimental data and theoretical arguments presented have to describe the three possible general outcomes predicted for the behavior of the temporal framework of systems consolidation – acceleration, “maintenance” or delay – after being triggered by contextual degree of novelty and/or the kind of mnemonic process engaged (consolidation, retrieval, reconsolidation) in the light of the putative background of the synaptic occupancy/reset hypothesis: as shown in the second and third lines below, each trigger factor results in a different impact upon the number of available synapses, which, on its turn, recruits a different mechanism that leads to the observed temporal effects. The novelty here – and it is hopefully a *testable possibility –* is that we propose two different backgrounds, plasticity taking place upon a **fixed** versus a **variable number of available synapses in the CA1 area**.

*First/new learning plasticity* would have to produce its engrammatic embodiment working with a **fixed subset of available synapses**, with plasticity directed to select and connect to specific neocortical target areas establishing a memory trace, while other, *reactivation-induced plasticities* would be able to somehow induce an **increase in the total number of synapses in the variable subset of available synapses**, at least within certain limits. The cognitive process induced by reactivation studied above was mainly reconsolidation (De Oliveira Alvares et al., 2012, 2013; Cassini et al., 2013; Sierra et al., 2013; Crestani et al., 2015; Haubrich et al., 2015), but processes mobilizing a change in the number of available synapses may also include extinction (Bouton and Moody, 2004; Sotres-Bayon et al., 2006; Myskiw and Izquierdo, 2012; Cassini et al., 2013, 2017; Sierra et al., 2017; Haubrich et al., 2017) and possibly even the intermediary category known as subsequent learnings (Tayler et al., 2011; Crestani and Quillfeldt, 2016; Crestani et al., 2018a,b). Of course non-reactivating, *plain retrieval* is proposed as a process as inert as the consequence of a cognitively poor, uneventful life (as criticized, e.g., by Neisser, 1967): in these cases, an automatic reset would take place at regular intervals, acting as a putative maintenance mechanism.

## Memories Are Synaptic Pathways: Testing the Hypothesis

In the natural sciences – as mentioned before – theories are measured not only by their *explanatory* power and *testability*, but a good *predictive* capacity is also desired (Bunge, 1967, 1985). Besides, theories are no better than the experimental data available in their support. Here, the challenge is being able to directly *probe the engram*, a concept originally introduced by Richard Semon (Semon, 1904; Josselyn et al., 2017), and considered intractable until the recent emergence of new, powerful recording and labeling technological tools, such as optogenetics (Cowansage et al., 2014; Nabavi et al., 2014; Tonegawa et al., 2015a,b; Cai et al., 2016; Rashid et al., 2016) and chemogenetics (Roth, 2016; Atasoy and Sternson, 2018; Campbell and Marchant, 2018; Muir et al., 2018), usually combined with multineuronal recordings (Sakurai et al., 2018). Optogenetics, outstanding and promising as it is (Goshen, 2014; Bickle, 2016), still has to evolve as a methodology considering the technical limitations that reduce their interpretability (see Baxter and Croxson, 2013; Hardt and Nadel, 2018). Even systems consolidation has been approached with this technique (Doron and Goshen, 2017), despite some arguable preliminary findings (Kitamura et al., 2017).

In order to empirically test SORT, first we need an operational definition of *engram.* Most authors think on the engram as an *object* to be found, something that is stored *in* a particular brain structure, but, since what neural plasticity allows – in order to record learning – is the establishment of a new neural network that represents the experience, **it is much more interesting and productive to define the engram as the organizational pattern *of* that very same brain structure** – something already hinted by Pavlov (1904). Thus, an engram would be the full established neural network consisting of a collection of *multiple pathways that connects/binds*, end-to-end, S to M, i.e., the **S**ensory (or internal) input signals with the adequate **M**otor outputs – in order to allow that the right stimuli “lead” to the correct, learned response behavior. This would be the basic engram, a tripartite structure selected along animal evolution, analogous to some extent to an innate behavior circuitry (see Fuster, 1995, 1997). Additional considerations could, without much difficulty, accommodate other kinds of non-sensory inputs, such as internally generated signals, as well as non-motor outputs, including those involving imaginary or even abstract thoughts, as humans can do.

**If its true that memory traces are the neural pathways, selected and marked by plasticity at the dendritic spines level, that c*onnect sensory inputs to motor outputs to produce a learned response*, the direct observation of synapses in activity might be more informative than recording whole-cell activity, as many existing techniques already permit**. Observing just at the neuronal level may incur in interpretation difficulties since, in principle, there might exist many different ways to connect the same set of neurons to obtain one same response pattern, which would lead to false positives. Another limitation derives from the fact that hippocampal neurons are known multitaskers, i.e., they simultaneously participate in different functions running in parallel, e.g., working as place cell as well as a engram-recording agent (Knierim, 2015; Lisman, 2017). Hippocampal indexes would consist of the exact relay connections necessary to store the memory/engram of one experience as a distributed network established over the large, sparse neocortical circuitry.

Thus, a subset of HPC CA1 pyramidal cells might collectively establish working connections with afferences pointing to the very same set of neocortical areas that have initially received and processed every sensory or multimodal information produced by the learning experience and conveyed to the HPC for integration and storage (Teyler and DiScenna, 1986). Of course this would *not* be a direct projection (however some might), since the efference must be relayed by at least two intermediary stations – entorhinal and perirhinal cortices – before effectively reaching neocortical targets. The difficulties of these necessarily tortuous pathways will not be elaborated here, and remains unknown. However this multilevel, stepped process resembles some classical multilayered connectionist models (Marr, 1971; Leng et al., 1994; McClelland et al., 1995; Sardesai et al., 2001), which might provide a hint to explain how a relatively small set of hippocampal cells might reach and control what is supposed to be a large number of cortical cells: studying the organization of these pathways, layer to layer, might help to understand how it works.

SORT proposes that CA1 cells establish strong synapses with previously available, yet not connected efferent axons projecting to the cortex, thus recruiting the exact set of cortical components that compose the engram. It is reasonable to consider that a direct test of this hypothesis is not technically feasible right now, at least not employing present optogenetic tools that lack resolution both in the spatial (diffusion covers only small volumes) and the temporal (fast on/off control might not reflect the longer temporal framework necessary for plastic changes to take place) domains, among other limitations (Baxter and Croxson, 2013; Kim et al., 2016; Hardt and Nadel, 2018; Sakurai et al., 2018). Thus, to visualize the whole set of CA1 (pyramidal cells’) plastic synapses that might specifically be involved in connecting to those axonal efferences projecting to NCTX, it may be much more adequate to employ *fluorescent microscopy.* These techniques allow for the imaging of *dendritic spine dynamics* itself, and include confocal laser scanning, transcranial two/multi-photon microscopes, or fiber-optics endomicroscopy (Maiti et al., 2015; Sakurai et al., 2018). They have already been proven efficient to image hippocampal place cells activity in subcellular resolution during navigation (Dombeck et al., 2010), and even to study long-term memory related neural ensemble activity in the amygdala (Grewe et al., 2017). Calcium-imaging techniques such as Cal-Light or FLARE, might allow a functional readout of synaptic activity in near real time and with great spatial resolution (Sakaguchi and Hayashi, 2012). The biggest challenge would be to identify, follow and control tridimensional synaptic patterns that take place over sparse populations of neurons in a brain area, but this might be the path to meet at least the first of Mayford’s experimental criteria to “definitely identify an engram for a declarative memory”: “identify a learning-induced molecular and corresponding functional cellular change in a specific subset of neurons” (Mayford, 2013): the other three criteria – basically three different ways to tamper with engrams – might then follow easily. Only after this, we might consider further investigating, for instance, the nature of the putative different *levels of resetability*, that might possibly be consequence of different neurochemical and morphofunctional properties among synapse populations I, II, and III, e.g., the presence of a molecular “safeguard” system, differences hopefully detectable: clearly, population III (quasi-stable or the “episodic” memory subset) would be the most “safeguarded” of them, which doesn’t mean it can’t somehow be reset – erased, a fate not even the best episodic memories cannot escape.

Until a few years ago, the memory research field was subject to conform to William James’s cautionary advice that “the only proof of there being retention is that recall actually takes place” (James, 1890, chapter XVI), since there was no possible way to “grasp” any material trait of “memory” to be examined: the nature of the engram has been clearly beyond the reach of most available technologies. In this aspect resided the great strength of behavioral neuroscience to this day, i.e., to be the only tool that allow “peeping” into the engram, whatever be its nature, actually “seeing the invisible.” The golden standard to confirm the presence of a memory trace, however, may never cease to be basically behavioral, but whatever the real nature of the engram is, it must accommodate not only the well-described plasticity machinery underlying synaptic consolidation, but also all the essential properties behaviorally observed, such as (1) the capacity of being constantly updated by reconsolidation, (2) the slow, complex process of consolidation at the system levels, and (3) the diversity of memory systems that exist in the mammalian brain. We may still be far from a full understanding of such a complex material entity, but technological advances seem to be pushing us toward some light (Josselyn et al., 2015; Poo et al., 2016; Queenan et al., 2017).

Understanding the nature of the physical trace of memory, or process that allows experience-based behavior modification in animals, is not only important as basic knowledge, but has also practical/clinical relevance, considering how devastating pathologies of memory may be. However, as is typical in technology nowadays, any further development must be preceded by a better understanding of the basic science behind the phenomenon, which benefits immensely from some theoretical elaboration.

## Ethics Statement

The paper is a conceptual review discussing different experimental works and no new experiment was performed.

## Author Contributions

JQ is the sole author of this paper and participated in all processes of writing this manuscript. In addition, he participated in many of the manuscripts reviewed, having generated the integrative hypothesis presented in this manuscript.

## Conflict of Interest Statement

The author declares that the research was conducted in the absence of any commercial or financial relationships that could be construed as a potential conflict of interest.

## Abbreviations

ACC: anterior cingulate cortex
CA1: *Cornu Ammonis 1* area of the hippocampus
CA3: *Cornu Ammonis 3* area of the hippocampus
CFC: contextual fear conditioning
CTT: *Competitive Trace Theory*
DG: dentate gyrus area of the hippocampus
DRT: *Distributed Reinstatement Theory*
ERC: entorhinal cortex
GluN2B: subunit N2B of NMDA receptor
HIT: Hippocampal Memory Indexing Theory
HPC: hippocampus
HSAM: highly superior autobiographical memory
IP: “intermediary plexus” – the neural network between S and M
LGVCCs: L-type voltage-gated calcium channels
M: motor outputs
MEMSET: extra-navigational *Memory Set of Functions*
mPFC: medial prefrontal cortex
MTL: medial temporal lobe
MTT: Multiple Trace Theory
NAVSET: Navigational Set of Functions
NCTX: neocortex
PPC: posterior parietal cortex
PTSD: post-traumatic stress disorder
RA: retrograde amnesia
S: sensory inputs
SMSC: Standard Model of Memory (Systems) Consolidation
TTT: *Trace Transformation Theory*

## References

[B1] Agustina López M. Jimena Santos M. Cortasa S. Fernández R. S. Carbó Tano M. Pedreira M. E. (2016). Different dimensions of the prediction error as a decisive factor for the triggering of the reconsolidation process. *Neurobiol. Learn. Mem.* 136 210–219. 10.1016/j.nlm.2016.10.016 27815213

[B2] Alberini C. M. (2011). The role of reconsolidation and the dynamic process of long-term memory formation and storage. *Front. Behav. Neurosci.* 5:12. 10.3389/fnbeh.2011.00012 21436877 PMC3056265

[B3] Anokhin K. V. Tiunova A. A. Rose S. P. (2002). Reminder effects - reconsolidation or retrieval deficit? Pharmacological dissection with protein synthesis inhibitors following reminder for a passive-avoidance task in young chicks. *Eur. J. Neurosci.* 15 1759–1765. 10.1046/j.1460-9568.2002.02023.x 12081655

[B4] Atasoy D. Sternson S. M. C. (2018). Chemogenetic tools for causal cellular and neuronal biology. *Physiol. Rev.* 98 391–418. 10.1152/physrev.00009.2017 29351511 PMC5866359

[B5] Bartlett F. C. (1932). *Remembering - a Study in Experimental and Social Psychology.* Cambridge: Cambridge University Press.

[B6] Baxter M. G. Croxson P. L. (2013). Behavioral control by the orbital prefrontal cortex: reversal of fortune. *Nat. Neurosci.* 16 984–985. 10.1038/nn.3472 23887130

[B7] Beeman C. L. Bauer P. S. Pierson J. L. Quinn J. J. (2013). Hippocampus and medial prefrontal cortex contributions to trace and contextual fear memory expression over time. *Learn. Mem.* 20 336–343. 10.1101/lm.031161.113 23685809

[B8] Besnard A. Sahay A. (2016). Adult hippocampal neurogenesis, fear generalization, and stress. *Neuropsychopharmacology* 41 24–44. 10.1038/npp.2015.167 26068726 PMC4677119

[B9] Bianchin M. Walz R. Ruschel A. C. Zanatta M. S. Da Silva R. C. Bueno e Silva M. (1993). Memory expression is blocked by the infusion of CNQX into the hippocampus and/or the amygdala up to 20 days after training. *Behav. Neural Biol.* 59 83–86. 10.1016/0163-1047(93)90782-D 8476386

[B10] Bickle J. (2016). Revolutions in neuroscience: tool development. *Front. Syst. Neurosci.* 10:24 10.3389/fnsys.2016.00024 PMC4782158 27013992

[B11] Biedenkapp J. C. Rudy J. W. (2007). Context pre-exposure prevents forgetting of a contextual fear memory: implication for regional changes in brain activation patterns associated with recent and remote memory tests. *Learn. Mem.* 14 200–203. 10.1101/lm.499407 17351145 PMC2519802

[B12] Bouton M. E. Moody E. W. (2004). Memory processes in classical conditioning. *Neurosci. Biobehav. Rev.* 28 663–674. 10.1016/j.neubiorev.2004.09.001 15555676

[B13] Braitenberg V. Schütz A. (1983). “Some anatomical comments on the hippocampus,” in *Neurobiology of the Hippocampus*, ed. Seifert W. (London: Academic Press), 21–37.

[B14] Broadbent N. J. Clark R. E. (2013). Remote context fear conditioning remains hippocampus-dependent irrespective of training protocol, training-surgery interval, lesion size, and lesion method. *Neurobiol. Learn. Mem.* 106 300–308. 10.1016/j.nlm.2013.08.008 23994542

[B15] Bull E. Whittington M. (2007). “Local circuits,” in *The Hippocampus Book* Vol. 8 eds Andersen P. Morris R. Amaral D. Bliss T. O’Keefe J. (New York, NY: Oxford University Press), 297–320.

[B16] Bunge M. (1967). *Scientific Research. Strategy and Philosophy.* New York, NY: Springer-Verlag.

[B17] Bunge M. (1985). *Epistemology & Methodology III: Philosophy of Science and Technology Part I: Formal and Physical Sciences (Treatise on Basic Philosophy, part I)*, Vol. 7 Dordrecht: D. Reidel Publ. Co, 28.

[B18] Bunge M. (2010). *Matter and Mind: A Philosophical Inquiry.* Dordrecht: Springer 10.1007/978-90-481-9225-0

[B19] Bustos S. G. Giachero M. Maldonado H. Molina V. A. (2010). Previous stress attenuates the susceptibility to Midazolam’s disruptive effect on fear memory reconsolidation: influence of pre-reactivation D-cycloserine administration. *Neuropsychopharmacology* 35 1097–1108. 10.1038/npp.2009.215 20043007 PMC3055408

[B20] Bustos S. G. Maldonado H. Molina V. A. (2009). Disruptive effect of midazolam on fear memory reconsolidation: decisive influence of reactivation time span and memory age. *Neuropsychopharmacology* 34 446–457. 10.1038/npp.2008.75 18509330

[B21] Buzsáki G. (1996). *Rhythms of the Brain.* New York, NY: Oxford University Press.

[B22] Cai D. J. Aharoni D. Shuman T. Shobe J. Biane J. Song W. (2016). A shared neural ensemble links distinct contextual memories encoded close in time. *Nature* 534 115–118. 10.1038/nature17955 27251287 PMC5063500

[B23] Campbell E. J. Marchant N. J. (2018). The use of chemogenetics in behavioural neuroscience: receptor variants, targeting approaches and caveats. *Br. J. Pharmacol.* 175 994–1003. 10.1111/bph.14146 29338070 PMC5843707

[B24] Cassini L. F. Flavell C. R. Amaral O. B. Lee J. L. C. (2017). On the transition from reconsolidation to extinction of contextual fear memories. *Learn. Mem.* 24 392–399. 10.1101/lm.045724.117 28814464 PMC5580521

[B25] Cassini L. F. Sierra R. O. Haubrich J. Crestani A. P. Santana F. de Oliveira Alvares L. (2013). Memory reconsolidation allows the consolidation of a concomitant weak learning through a synaptic tagging and capture mechanism. *Hippocampus* 23 931–941. 10.1002/hipo.22149 23733489

[B26] Cipolotti L. Bird C. M. (2006). Amnesia and the hippocampus. *Curr. Opin. Neurol.* 19 593–598. 10.1097/01.wco.0000247608.42320.f9 17102699

[B27] Clem R. L. Huganir R. L. (2010). Calcium-permeable AMPA receptor dynamics mediate fear memory erasure. *Science* 330 1108–1112. 10.1126/science.1195298 21030604 PMC3001394

[B28] Cohen N. J. (1981). Neuropsychological evidence for a distinction between procedural and declarative knowledge in human memory and amnesia. *Diss. Abstr. Int.* 41(12–B, Pt 1):4733.

[B29] Cohen N. J. Squire L. R. (1980). Preserved learning and retention of pattern-analyzing skill in amnesia: dissociation of knowing how and knowing that. *Science* 210 207–210. 10.1126/science.7414331 7414331

[B30] Cowansage K. K. Shuman T. Dillingham B. C. Chang A. Golshani P. Mayford M. (2014). Direct reactivation of a coherent neocortical memory of context. *Neuron* 84 432–441. 10.1016/j.neuron.2014.09.022 25308330 PMC4372249

[B31] Craik F. I. (2002). Levels of processing: past, present. and future? *Memory* 10 305–318. 10.1080/09658210244000135 12396643

[B32] Crestani A. P. Krueger J. N. Barragan E. V. Nakazawa Y. Nemes S. E. Quillfeldt J. A. (2018a). Metaplasticity contributes to memory formation in the hippocampus. *Neuropsychopharmacology* 44 408–414. 10.1038/s41386-018-0096-7 29849054 PMC6300591

[B33] Crestani A. P. Sierra R. O. Machado A. Haubrich J. Scienza K. M. de Oliveira Alvares L. (2018b). Hippocampal plasticity mechanisms mediating experience-dependent learning change over time. *Neurobiol. Learn. Mem.* 150 56–63. 10.1016/j.nlm.2018.02.020 29501525

[B34] Crestani A. P. Quillfeldt J. A. (2016). Can previous learning alter future plasticity mechanisms? *Behav. Neurosci.* 130 1–5. 10.1037/bne0000122 26795579

[B35] Crestani A. P. Zacouteguy Boos F. Haubrich J. Ordoñez Sierra R. Santana F. Molina J. M. (2015). Memory reconsolidation may be disrupted by a distractor stimulus presented during reactivation. *Sci. Rep.* 5:13633. 10.1038/srep13633 26328547 PMC4556962

[B36] De Oliveira Alvares L. Crestani A. P. Cassini L. F. Haubrich J. Santana F. Quillfeldt J. A. (2013). Reactivation enables memory updating, precision-keeping and strengthening: exploring the possible biological roles of reconsolidation. *Neuroscience* 244 42–48. 10.1016/j.neuroscience.2013.04.005 23587841

[B37] De Oliveira Alvares L. Einarsson E. Ö. Santana F. Crestani A. P. Haubrich J. Cassini L. F. (2012). Periodically reactivated context memory retains its precision and dependence on the hippocampus. *Hippocampus* 22 1092–1095. 10.1002/hipo.20983 22120981

[B38] De Oliveira Alvares L. Genro B. P. Diehl F. Quillfeldt J. A. (2008a). Differential role of the hippocampal endocannabinoid system in the memory consolidation and retrieval mechanisms. *Neurobiol. Learn. Mem.* 90 1–9. 10.1016/j.nlm.2008.01.009 18342551

[B39] De Oliveira Alvares L. Pasqualini Genro B. Diehl F. Molina V. A. Quillfeldt J. A. (2008b). Opposite action of hippocampal CB1 receptors in memory reconsolidation and extinction. *Neuroscience* 154 1648–1655. 10.1016/j.neuroscience.2008.05.005 18554811

[B40] Debiec J. LeDoux J. E. Nader K. (2002). Cellular and systems reconsolidation in the hippocampus. *Neuron* 36 527–538. 10.1016/S0896-6273(02)01001-2 12408854

[B41] Ding H. K. Teixeira C. M. Frankland P. W. (2008). Inactivation of the anterior cingulate cortex blocks expression of remote, but not recent, conditioned taste aversion memory. *Learn. Mem.* 15 290–293. 10.1101/lm.905008 18441286

[B42] Dombeck D. A. Harvey C. D. Tian L. Looger L. L. Tank D. W. (2010). Functional imaging of hippocampal place cells at cellular resolution during virtual navigation. *Nat. Neurosci.* 13 1433–1440. 10.1038/nn.2648 20890294 PMC2967725

[B43] Doron A. Goshen I. (2017). Investigating the transition from recent to remote memory using advanced tools. *Brain Res. Bull.* 141 35–43. 10.1016/j.brainresbull.2017.09.005 28939475

[B44] Dudai Y. (1996). Consolidation: fragility on the road to the engram. *Neuron* 17 367–370. 10.1016/S0896-6273(00)80168-3 8816699

[B45] Duvarci S. Nader K. (2004). Characterization of fear memory reconsolidation. *J. Neurosci.* 24 9269–9275. 10.1523/JNEUROSCI.2971-04.2004 15496662 PMC6730081

[B46] Einarsson E. Ö. Pors J. Nader K. (2015). Systems reconsolidation reveals a selective role for the anterior cingulate cortex in generalized contextual fear memory expression. *Neuropsychopharmacology* 40 480–487. 10.1038/npp.2014.197 25091528 PMC4443963

[B47] Fernández R. S. Boccia M. M. Pedreira M. E. (2016). The fate of memory: reconsolidation and the case of Prediction Error. *Neurosci. Biobehav. Rev.* 68 423–441. 10.1016/j.neubiorev.2016.06.004 27287939

[B48] Ferreira A. R. Fürstenau L. Blanco C. Kornisiuk E. Sánchez G. Daroit D. (2003). Role of hippocampal M1 and M4 muscarinic receptor subtypes in memory consolidation in the rat. *Pharmacol. Biochem. Behav.* 74 411–415. 10.1016/S0091-3057(02)01007-9 12479962

[B49] Ferreira R. C. Medina J. H. Izquierdo I. (1992a). Late posttraining memory processing by entorhinal cortex: involvement of NMDA and GABAergic receptors. *Pharmacol. Biochem. Behav.* 41 767–771. 10.1016/0091-3057(92)90225-5 1350684

[B50] Ferreira M. B. Wolfman C. Walz R. Da Silva R. C. Zanatta M. S. Medina J. H. (1992b). NMDA-receptor-dependent, muscimol-sensitive role of the entorhinal cortex in post-training memory processing. *Behav. Pharmacol.* 3 387–391. 11224141

[B51] Frankland P. W. Bontempi B. (2005). The organization of recent and remote memories. *Nat. Rev. Neurosci.* 6 119–130. 10.1038/nrn1607 15685217

[B52] Frankland P. W. Bontempi B. Talton L. E. Kaczmarek L. Silva A. J. (2004a). The involvement of the anterior cingulate cortex in remote contextual fear memory. *Science* 304 881–883. 15131309 10.1126/science.1094804

[B53] Frankland P. W. Josselyn S. A. Anagnostaras S. G. Kogan J. H. Takahashi E. Silva A. J. (2004b). Consolidation of CS and US representations in associative fear conditioning. *Hippocampus* 14 557–569. 15301434 10.1002/hipo.10208

[B54] Fuster J. M. (1995). Memory in the cortex of the primate. *Biol. Res.* 28 59–72. 8728821

[B55] Fuster J. M. (1997). Network memory. *Trends Neurosci.* 20 451–459. 10.1016/S0166-2236(97)01128-4 9347612

[B56] Galotti K. M. (2018). *Cognitive Psychology In and Out of the Laboratory*, 6th Edn, Vol. 1 Thousand Oaks, CA: SAGE Publications, Inc, 2–26

[B57] Ghosh V. E. Gilboa A. (2014). What is a memory schema? A historical perspective on current neuroscience literature. *Neuropsychologia* 53 104–114. 10.1016/j.neuropsychologia.2013.11.010 24280650

[B58] Goshen I. (2014). The optogenetic revolution in memory research. *Trends Neurosci.* 37 511–522. 10.1016/j.tins.2014.06.002 25022518

[B59] Goshen I. Brodsky M. Prakash R. Wallace J. Gradinaru V. Ramakrishnan C. (2011). Dynamics of retrieval strategies for remote memories. *Cell* 147 678–689. 10.1016/j.cell.2011.09.033 22019004

[B60] Gould E. Beylin A. Tanapat P. Reeves A. Shors T. J. (1999a). Learning enhances adult neurogenesis in the hippocampal formation. *Nat. Neurosci.* 2 260–265. 10195219 10.1038/6365

[B61] Gould E. Tanapat P. Hastings N. B. Shors T. J. (1999b). Neurogenesis in adulthood: a possible role in learning. *Trends Cogn. Sci.* 3 186–192. 10.1016/S1364-6613(99)01310-8 10322475

[B62] Graf P. Schacter D. L. (1985). Implicit and explicit memory for new associations in normal and amnesic subjects. *J. Exp. Psychol. Learn. Mem. Cogn.* 11 501–518. 10.1037/0278-7393.11.3.501 3160813

[B63] Grewe B. F. Gründemann J. Kitch L. J. Lecoq J. A. Parker J. G. Marshall J. D. (2017). Neural ensemble dynamics underlying a long-term associative memory. *Nature* 543 670–675. 10.1038/nature21682 28329757 PMC5378308

[B64] Han J. H. Kushner S. A. Yiu A. P. Cole C. J. Matynia A. Brown R. A. (2007). Neuronal competition and selection during memory formation. *Science* 316 457–460. 10.1126/science.1139438 17446403

[B65] Han J. H. Kushner S. A. Yiu A. P. Hsiang H. L. Buch T. Waisman A. (2009). Selective erasure of a fear memory. *Science* 323 1492–1496. 10.1126/science.1164139 19286560

[B66] Hardt O. Einarsson E. O. Nader K. (2010). A bridge over troubled water: reconsolidation as a link between cognitive and neuroscientific memory research traditions. *Annu. Rev. Psychol.* 61 141–167. 10.1146/annurev.psych.093008.100455 19575608

[B67] Hardt O. Nadel L. (2018). Systems consolidation revisited, but not revised: the promise and limits of optogenetics in the study of memory. *Neurosci. Lett.* 680 54–59. 10.1016/j.neulet.2017.11.062 29203208

[B68] Hasselmo M. E. (2006). The role of acetylcholine in learning and memory. *Curr. Opin. Neurobiol.* 16 710–715. 10.1016/j.conb.2006.09.002 17011181 PMC2659740

[B69] Hasselmo M. E. Stern C. E. (2018). A network model of behavioural performance in a rule learning task. *Philos. Trans. R. Soc. Lond. B Biol. Sci.* 373:20170275. 10.1098/rstb.2017.0275 29483357 PMC5832697

[B70] Haubrich J. Cassini L. F. Diehl F. Santana F. Fürstenau de Oliveira L. de Oliveira Alvares L. (2016). Novel learning accelerates systems consolidation of a contextual fear memory. *Hippocampus* 26 924–932. 10.1002/hipo.22575 26860633

[B71] Haubrich J. Crestani A. P. Cassini L. F. Santana F. Sierra R. O. Alvares Lde O. (2015). Reconsolidation allows fear memory to be updated to a less aversive level through the incorporation of appetitive information. *Neuropsychopharmacology* 40 315–326. 10.1038/npp.2014.174 25027331 PMC4443944

[B72] Haubrich J. Machado A. Boos F. Z. Crestani A. P. Sierra R. O. Alvares L. O. (2017). Enhancement of extinction memory by pharmacological and behavioral interventions targeted to its reactivation. *Sci. Rep.* 7:10960. 10.1038/s41598-017-11261-6 28887561 PMC5591313

[B73] Haubrich J. Nader K. (2018). “Memory reconsolidation,” in *Behavioral Neuroscience of Learning and Memory. Current Topics in Behavioral Neurosciences* Vol. 37 eds Clark R. E. Martin S. (Cham: Springer), 151–176. 10.1007/7854_2016_463 27885549

[B74] Hupbach A. Hardt O. Gomez R. Nadel L. (2008). The dynamics of memory: context-dependent updating. *Learn. Mem.* 15 574–579. 10.1101/lm.1022308 18685148

[B75] Insel N. Takehara-Nishiuchi K. (2013). The cortical structure of consolidated memory: a hypothesis on the role of the cingulate-entorhinal cortical connection. *Neurobiol. Learn. Mem.* 106 343–350. 10.1016/j.nlm.2013.07.019 23911917

[B76] Izquierdo I. Bianchin M. Silva M. B. Zanatta M. S. Walz R. Ruschel A. C. (1993a). CNQX infused into rat hippocampus or amygdala disrupts the expression of memory of two different tasks. *Behav. Neural Biol.* 59 1–4. <> 8095135 10.1016/0163-1047(93)91061-q

[B77] Izquierdo I. Medina J. H. Bianchin M. Walz R. Zanatta M. S. Da Silva R. C. (1993b). Memory processing by the limbic system: role of specific neurotransmitter systems. *Behav. Brain Res.* 58 91–98. 10.1016/0166-4328(93)90093-6 7907882

[B78] Izquierdo I. Quillfeldt J. A. Zanatta M. S. Quevedo J. Schaeffer E. Schmitz P. K. (1997). Sequential role of hippocampus and amygdala, entorhinal cortex and parietal cortex in formation and retrieval of memory for inhibitory avoidance in rats. *Eur. J. Neurosci.* 9 786–793. 10.1111/j.1460-9568.1997.tb01427.x 9153585

[B79] James W. (1890). *The Principles of Psychology.* New York, NY: Henry Holt and Company.

[B80] Jasnow A. M. Lynch J. F. III Gilman T. L. Riccio D. C. (2017). Perspectives on fear generalization and its implications for emotional disorders. *J. Neurosci. Res.* 95 821–835. 10.1002/jnr.23837 27448175

[B81] Jerusalinsky D. Ferreira M. B. Walz R. Da Silva R. C. Bianchin M. Ruschel A. C. (1992). Amnesia by post-training infusion of glutamate receptor antagonists into the amygdala, hippocampus, and entorhinal cortex. *Behav. Neural Biol.* 58 76–80. 10.1016/0163-1047(92)90982-A 1417675

[B82] Jerusalinsky D. Kornisiuk E. Alfaro P. Quillfeldt J. Alonso M. Verde E. R. (1998). Muscarinic toxin selective for m4 receptors impairs memory in the rat. *Neuroreport* 9 1407–1411. 10.1097/00001756-199805110-00029 9631438

[B83] Jerusalinsky D. Quillfeldt J. A. Walz R. Da Silva R. C. Bueno e Silva M. Bianchin M. (1994). Effect of the infusion of the GABA-A receptor agonist, muscimol, on the role of the entorhinal cortex, amygdala, and hippocampus in memory processes. *Behav. Neural Biol.* 61 132–138. 10.1016/S0163-1047(05)80066-4 7911300

[B84] Josselyn S. A. Frankland P. W. (2018). Memory allocation: mechanisms and function. *Annu. Rev. Neurosci.* 41 389–413. 10.1146/annurev-neuro-080317-061956 29709212 PMC9623596

[B85] Josselyn S. A. Köhler S. Frankland P. W. (2015). Finding the engram. *Nat. Rev. Neurosci.* 16 521–534. 10.1038/nrn4000 26289572

[B86] Josselyn S. A. Köhler S. Frankland P. W. (2017). Heroes of the engram. *J. Neurosci.* 37 4647–4657. 10.1523/JNEUROSCI.0056-17.2017 28469009 PMC6596490

[B87] Kim J. I. Cho H. Y. Han J. H. Kaang B. K. (2016). Which neurons will be the engram – activated neurons and/or more excitable neurons? *Exp. Neurobiol.* 25 55–63. 10.5607/en.2016.25.2.55 27122991 PMC4844563

[B88] Kim J. J. Fanselow M. S. (1992). Modality-specific retrograde amnesia of fear. *Science* 256 675–677. 10.1126/science.1585183 1585183

[B89] Kitamura T. Ogawa S. K. Roy D. S. Okuyama T. Morrissey M. D. Smith L. M. (2017). Engrams and circuits crucial for systems consolidation of a memory. *Science* 356 73–78. 10.1126/science.aam6808 28386011 PMC5493329

[B90] Kitamura T. Saitoh Y. Takashima N. Murayama A. Niibori Y. Ageta H. (2009). Adult neurogenesis modulates the hippocampus-dependent period of associative fear memory. *Cell* 139 814–827. 10.1016/j.cell.2009.10.020 19914173

[B91] Knierim J. J. (2015). From the GPS to HM: place cells, grid cells, and memory. *Hippocampus* 25 719–725. 10.1002/hipo.22453 25788454

[B92] Krawczyk M. C. Fernández R. S. Pedreira M. E. Boccia M. M. (2017). Toward a better understanding on the role of prediction error on memory processes: from bench to clinic. *Neurobiol. Learn. Mem.* 142(Pt A), 13–20. 10.1016/j.nlm.2016.12.011 28017817

[B93] Lavenex P. Amaral D. G. (2000). Hippocampal-neocortical interaction: a hierarchy of associativity. *Hippocampus* 10 420–430. 10.1002/1098-1063(2000)10:4<420::AID-HIPO8>3.0.CO;2-5 10985281

[B94] Lee J. L. (2010). Memory reconsolidation mediates the updating of hippocampal memory content. *Front. Behav. Neurosci.* 4:168. 10.3389/fnbeh.2010.00168 21120142 PMC2991235

[B95] Lee J. L. Milton A. L. Everitt B. J. (2006). Reconsolidation and extinction of conditioned fear: inhibition and potentiation. *J. Neurosci.* 26 10051–10056. 10.1523/JNEUROSCI.2466-06.2006 17005868 PMC6674482

[B96] Leng X. McGrann J. V. Quillfeldt J. A. Shaw G. L. Shenoy K. V. (1994). *Learning and Memory Processes and the Modularity of the Brain. Neural Bases of Learning and Memory*, ed. Delacour J. (Singapore: World Scientific Press).

[B97] LePort A. K. Mattfeld A. T. Dickinson-Anson H. Fallon J. H. Stark C. E. Kruggel F. (2012). Behavioral and neuroanatomical investigation of Highly Superior Autobiographical Memory (HSAM). *Neurobiol. Learn. Mem.* 98 78–92. 10.1016/j.nlm.2012.05.002 22652113 PMC3764458

[B98] LePort A. K. Stark S. M. McGaugh J. L. Stark C. E. (2017). A cognitive assessment of highly superior autobiographical memory. *Memory* 25 276–288. 10.1080/09658211.2016.1160126 26982996 PMC5488704

[B99] Lewis D. J. (1979). Psychobiology of active and inactive memory. *Psychol. Bull.* 86 1054–1083. 10.1037/0033-2909.86.5.1054 386401

[B100] Lisman J. (2017). Criteria for identifying the molecular basis of the engram (CaMKII, PKMzeta). *Mol. Brain* 10:55. 10.1186/s13041-017-0337-4 29187215 PMC5707903

[B101] Lopez J. Gamache K. Milo C. Nader K. (2018). Differential role of the anterior and intralaminar/lateral thalamic nuclei in systems consolidation and reconsolidation. *Brain Struct. Funct.* 223 63–76. 10.1007/s00429-017-1475-2 28710525

[B102] Maiti P. Manna J. McDonald M. P. (2015). Merging advanced technologies with classical methods to uncover dendritic spine dynamics: a hot spot of synaptic plasticity. *Neurosci. Res.* 96 1–13. 10.1016/j.neures.2015.02.007 25728560

[B103] Maren S. Aharonov G. Fanselow M. S. (1997). Neurotoxic lesions of the dorsal hippocampus and Pavlovian fear conditioning in rats. *Behav. Brain Res.* 88 261–274. 10.1016/S0166-4328(97)00088-0 9404635

[B104] Marr D. (1971). Simple memory: a theory for archicortex. *Philos. Trans. R. Soc. Lond. B Biol. Sci.* 262 23–81. 10.1098/rstb.1971.0078 4399412

[B105] Mayford M. (2013). The search for a hippocampal engram. *Philos. Trans. R. Soc. Lond. B Biol. Sci.* 369:20130161. 10.1098/rstb.2013.0161 24298162 PMC3843892

[B106] McClelland J. L. McNaughton B. L. O’Reilly R. C. (1995). Why there are complementary learning systems in the hippocampus and neocortex: insights from the successes and failures of connectionist models of learning and memory. *Psychol. Rev.* 102 419–457. 10.1037/0033-295X.102.3.419 7624455

[B107] McDonald R. J. White N. M. (1993). A triple dissociation of memory systems: hippocampus, amygdala, and dorsal striatum. *Behav. Neurosci.* 107 3–22. 10.1037/0735-7044.107.1.3 8447956

[B108] McNaughton B. L. Nadel L. (1990). “Hebb-Marr networks and the neurobiological representation of action in space,” in *Developments in Connectionist Theory. Neuroscience and Connectionist Theory*, eds Gluck M. A. Rumelhart D. E. (Hillsdale, NJ: Lawrence Erlbaum Associates, Inc), 1–63.

[B109] Moser M. B. Rowland D. C. Moser E. I. (2015). Place cells, grid cells, and memory. *Cold Spring Harb. Perspect. Biol.* 7:a021808. 10.1101/cshperspect.a021808 25646382 PMC4315928

[B110] Muir J. Lopez J. Bagot R. C. (2018). Wiring the depressed brain: optogenetic and chemogenetic circuit interrogation in animal models of depression. *Neuropsychopharmacology* 10.1038/s41386-018-0291-6 [Epub ahead of print]. 30555161 PMC6461994

[B111] Myskiw J. C. Izquierdo I. (2012). Posterior parietal cortex and long-term memory: some data from laboratory animals. *Front. Integr. Neurosci.* 6:8 10.3389/fnint.2012.00008 PMC3287050 22375107

[B112] Nabavi S. Fox R. Proulx C. D. Lin J. Y. Tsien R. Y. Malinow R. (2014). Engineering a memory with LTD and LTP. *Nature* 511 348–352. 10.1038/nature13294 24896183 PMC4210354

[B113] Nadel L. Campbell J. Ryan L. (2007). Autobiographical memory retrieval and hippocampal activation as a function of repetition and the passage of time. *Neural Plast.* 2007:90472. 10.1155/2007/90472 18274617 PMC2233815

[B114] Nadel L. Hardt O. (2011). Update on memory systems and processes. *Neuropsychopharmacology* 36 251–273. 10.1038/npp.2010.169 20861829 PMC3055510

[B115] Nadel L. Moscovitch M. (1997). Memory consolidation, retrograde amnesia and the hippocampal complex. *Curr. Opin. Neurobiol.* 7 217–227. 10.1016/S0959-4388(97)80010-4 9142752

[B116] Nadel L. Moscovitch M. (1998). Hippocampal contributions to cortical plasticity. *Neuropharmacology* 37 431–439. 10.1016/S0028-3908(98)00057-4 9704984

[B117] Nader K. Einarsson E. O. (2010). Memory reconsolidation: an update. *Ann. N. Y. Acad. Sci.* 1191 27–41. 10.1111/j.1749-6632.2010.05443.x 20392274

[B118] Nader K. Hardt O. (2009). A single standard for memory: the case for reconsolidation. *Nat. Rev. Neurosci.* 10 224–234. 10.1038/nrn2590 19229241

[B119] Nader K. Schafe G. E. LeDoux J. E. (2000a). Fear memories require protein synthesis in the amygdala for reconsolidation after retrieval. *Nature* 406 722–726. 10963596 10.1038/35021052

[B120] Nader K. Schafe G. E. LeDoux J. E. (2000b). The labile nature of consolidation theory. *Nat. Rev. Neurosci.* 1 216–219. 10.1038/35044580 11257912

[B121] Neisser U. (1967). *Cognitive Psychology.* New York, NY: Meredith Publishing Company.

[B122] Neisser U. Winograd E. (2006). *Remembering Reconsidered: Ecological and Traditional Approaches to the Study of Memory.* Cambridge: Cambridge University Press.

[B123] O’Keefe J. Nadel L. (1978). *The Hippocampus as a Cognitive Map.* Oxford: Oxford University Press.

[B124] O’Reilly R. C. Bhattacharyya R. Howard M. D. Ketz N. (2014). Complementary learning systems. *Cogn. Sci.* 38 1229–1248. 10.1111/j.1551-6709.2011.01214.x 22141588

[B125] Pavlov I. (1904). *Nobel Lecture. NobelPrize.org. Nobel Media AB 2019.* Available at: https://www.nobelprize.org/prizes/medicine/1904/pavlov/lecture/

[B126] Pedraza L. K. Sierra R. O. Boos F. Z. Haubrich J. Quillfeldt J. A. Alvares Lde O. (2016). The dynamic nature of systems consolidation: stress during learning as a switch guiding the rate of the hippocampal dependency and memory quality. *Hippocampus* 26 362–371. 10.1002/hipo.22527 26333109

[B127] Pitler T. A. Alger B. E. (1992). Cholinergic excitation of GABAergic interneurons in the rat hippocampal slice. *J. Physiol.* 450 127–142. 10.1113/jphysiol.1992.sp019119 1359121 PMC1176114

[B128] Poo M. M. Pignatelli M. Ryan T. J. Tonegawa S. Bonhoeffer T. Martin K. C. (2016). What is memory? The present state of the engram. *BMC Biol.* 14:40. 10.1186/s12915-016-0261-6 27197636 PMC4874022

[B129] Queenan B. N. Ryan T. J. Gazzaniga M. S. Gallistel C. R. (2017). On the research of time past: the hunt for the substrate of memory. *Ann. N. Y. Acad. Sci.* 1396 108–125. 10.1111/nyas.13348 28548457 PMC5448307

[B130] Quillfeldt J. A. De Oliveira Alvares L. (2015). “The hippocampal endocannabinoid system in different memory phases: unveiling the CA1 circuitry,” in *Cannabinoids and Modulation of Emotion, Memory, and Motivation*, eds Campolongo P. Fattore L. (New York, NY: Springer). 10.1007/978-1-4939-2294-9_3

[B131] Quillfeldt J. A. Schmitz P. K. Walz R. Bianchin M. Zanatta M. S. Medina J. H. (1994). CNQX infused into entorhinal cortex blocks memory expression, and AMPA reverses the effect. *Pharmacol. Biochem. Behav.* 48 437–440. 10.1016/0091-3057(94)90549-5 7522331

[B132] Quillfeldt J. A. Zanatta M. S. Schmitz P. K. Quevedo J. Schaeffer E. Lima J. B. (1996). Different brain areas are involved in memory expression at different times from training. *Neurobiol. Learn. Mem.* 66 97–101. 10.1006/nlme.1996.0050 8946402

[B133] Rasch B. Born J. (2013). About sleep’s role in memory. *Physiol. Rev.* 93 681–766. 10.1152/physrev.00032.2012 23589831 PMC3768102

[B134] Rashid A. J. Yan C. Mercaldo V. Hsiang H. L. Park S. Cole C. J. (2016). Competition between engrams influences fear memory formation and recall. *Science* 353 383–387. 10.1126/science.aaf0594 27463673 PMC6737336

[B135] Ribot T. (1881). *Les Maladies de la Mémoire.* Paris: Librairie Germer Balliere et. Cie.

[B136] Roediger H. L. De Soto K. A. (2015). “The psychology of reconstructive memory,” in *International Encyclopedia of the Social and Behavioral Sciences*, ed. Wright J. (New York, NY: Elsevier), 50–55. 10.1016/B978-0-08-097086-8.51016-2

[B137] Rolls E. T. (2017). A scientific theory of ars memoriae: spatial view cells in a continuous attractor. *Hippocampus* 27 570–579. 10.1002/hipo.22713 28176397

[B138] Rolls E. T. Kesner R. P. (2006). A computational theory of hippocampal function, and empirical tests of the theory. *Prog. Neurobiol.* 79 1–48. 10.1016/j.pneurobio.2006.04.005 16781044

[B139] Rolls E. T. Treves A. (1998). *Neural Networks and Brain Function.* New York, NY: Oxford University Press.

[B140] Rolls E. T. Treves A. Robertson R. G. Georges-François P. Panzeri S. (1998). Information about spatial view in an ensemble of primate hippocampal cells. *J. Neurophysiol.* 79 1797–1813. 10.1152/jn.1998.79.4.1797 9535949

[B141] Rose J. K. Rankin C. H. (2006). Blocking memory reconsolidation reverses memory-associated changes in glutamate receptor expression. *J. Neurosci.* 26 11582–11587. 10.1523/JNEUROSCI.2049-06.2006 17093079 PMC6674789

[B142] Roth B. L. (2016). DREADDs for neuroscientists. *Neuron* 89 683–694. 10.1016/j.neuron.2016.01.040 26889809 PMC4759656

[B143] Rudy J. W. (2008). Destroying memories to strengthen them. *Nat. Neurosci.* 11 1241–1242. 10.1038/nn1108-1241 18956008

[B144] Rudy J. W. (2009). Context representations, context functions, and the parahippocampal-hippocampal system. *Learn. Mem.* 16 573–585. 10.1101/lm.1494409 19794181 PMC2769166

[B145] Sakaguchi M. Hayashi Y. (2012). Catching the engram: strategies to examine the memory trace. *Mol. Brain* 5:32. 10.1186/1756-6606-5-32 22999350 PMC3462696

[B146] Sakurai Y. Osako Y. Tanisumi Y. Ishihara E. Hirokawa J. Manabe H. (2018). Multiple approaches to the investigation of cell assembly in memory research-present and future. *Front. Syst. Neurosci.* 12:21. 10.3389/fnsys.2018.00021 29887797 PMC5980992

[B147] Sánchez G. Alvares Lde O. Oberholzer M. V. Genro B. Quillfeldt J. da Costa J. C. (2009). M4 muscarinic receptors are involved in modulation of neurotransmission at synapses of Schaffer collaterals on CA1 hippocampal neurons in rats. *J. Neurosci. Res.* 87 691–700. 10.1002/jnr.21876 18816796

[B148] Santangelo V. Cavallina C. Colucci P. Santori A. Macrì S. McGaugh J. L. (2018). Enhanced brain activity associated with memory access in highly superior autobiographical memory. *Proc. Natl. Acad. Sci. U.S.A.* 115 7795–7800. 10.1073/pnas.1802730115 29987025 PMC6064994

[B149] Sara S. J. (2017). Sleep to remember. *J. Neurosci.* 37 457–463. 10.1523/JNEUROSCI.0297-16.2017 28100730 PMC6596760

[B150] Sardesai M. Figge C. Bodner M. Crosby M. Hansen J. Quillfeldt J. A. (2001). Reliable short-term memory in the trion model: toward a cortical language and grammar. *Biol. Cybern.* 84 173–182. 10.1007/s004220000204 11252635

[B151] Schon K. Atri A. Hasselmo M. E. Tricarico M. D. LoPresti M. L. Stern C. E. (2005). Scopolamine reduces persistent activity related to long-term encoding in the parahippocampal gyrus during delayed matching in humans. *J. Neurosci.* 25 9112–9123. 10.1523/JNEUROSCI.1982-05.2005 16207870 PMC6725748

[B152] Scoville W. B. Milner B. (1957). Loss of recent memory after bilateral hippocampal lesions. *J. Neurol. Neurosurg. Psychiatry* 20 11–21. 10.1136/jnnp.20.1.11 13406589 PMC497229

[B153] Sekeres M. J. Winocur G. Moscovitch M. (2018). The hippocampus and related neocortical structures in memory transformation. *Neurosci. Lett.* 680 39–53. 10.1016/j.neulet.2018.05.006 29733974

[B154] Semon R. (1904). “The mneme,” in *Engraphic Action of Stimuli on the Individual*, trans. Simon Louis (London: George Allen & Unwin).

[B155] Sherry D. Schacter D. (1987). The evolution of multiple memory systems. *Psychol. Rev.* 94 439–454. 10.1037//0033-295X.94.4.439

[B156] Shimizu E. Tang Y. P. Rampon C. Tsien J. Z. (2000). NMDA receptor-dependent synaptic reinforcement as a crucial process for memory consolidation. *Science* 290 1170–1174. 10.1126/science.290.5494.1170 11073458

[B157] Sierra R. O. Cassini L. F. Santana F. Crestani A. P. Duran J. M. Haubrich J. (2013). Reconsolidation may incorporate state-dependency into previously consolidated memories. *Learn. Mem.* 20 379–387. 10.1101/lm.030023.112 23782508

[B158] Sierra R. O. Pedraza L. K. Zanona Q. K. Santana F. Boos F. Z. Crestani A. P. (2017). Reconsolidation-induced rescue of a remote fear memory blocked by an early cortical inhibition: involvement of the anterior cingulate cortex and the mediation by the thalamic nucleus reuniens. *Hippocampus* 27 596–607. 10.1002/hipo.22715 28176459

[B159] Sotres-Bayon F. Cain C. K. LeDoux J. E. (2006). Brain mechanisms of fear extinction: historical perspectives on the contribution of prefrontal cortex. *Biol. Psychiatry* 60 329–336. 10.1016/j.biopsych.2005.10.012 16412988

[B160] Spiers H. J. Burgess N. Hartley T. Vargha-Khadem F. O’Keefe J. (2001a). Bilateral hippocampal pathology impairs topographical and episodic memory but not visual pattern matching. *Hippocampus* 11 715–725. 11811666 10.1002/hipo.1087

[B161] Spiers H. J. Maguire E. A. Burgess N. (2001b). Hippocampal amnesia. *Neurocase* 7 357–382. 10.1076/neur.7.5.357.16245 11744778

[B162] Spruston N. (2008). Pyramidal neurons: dendritic structure and synaptic integration. *Nat. Rev. Neurosci.* 9 206–221. 10.1038/nrn2286 18270515

[B163] Squire L. R. (2004). Memory systems of the brain: a brief history and current perspective. *Neurobiol. Learn. Mem.* 82 171–177. 10.1016/j.nlm.2004.06.005 15464402

[B164] Squire L. R. Alvarez P. (1995). Retrograde amnesia and memory consolidation: a neurobiological perspective. *Curr. Opin. Neurobiol.* 5 169–177. 10.1016/0959-4388(95)80023-9 7620304

[B165] Squire L. R. Bayley P. J. (2007). The neuroscience of remote memory. *Curr. Opin. Neurobiol.* 17 185–196. 10.1016/j.conb.2007.02.006 17336513 PMC2277361

[B166] Squire L. R. Cohen N. J. Nadel L. (1984). “The medial temporal region and memory consolidation: a new hypothesis,” in *Memory Consolidation: Psychobiology of Cognition*, eds Weingartner H. Parker E. S. (Hillsdale, NJ: Lawrence Erlbaum Associates), 185–210.

[B167] Stern C. E. Hasselmo M. E. (2005). Less is more: how reduced activity reflects stronger recognition. *Neuron* 47 625–627. 10.1016/j.neuron.2005.08.013 16129391

[B168] Sutherland R. J. O’Brien J. Lehmann H. (2008). Absence of systems consolidation of fear memories after dorsal, ventral, or complete hippocampal damage. *Hippocampus* 18 710–718. 10.1002/hipo.20431 18446823

[B169] Sutherland R. J. Sparks F. T. Lehmann H. (2010). Hippocampus and retrograde amnesia in the rat model: a modest proposal for the situation of systems consolidation. *Neuropsychologia* 48 2357–2369. 10.1016/j.neuropsychologia.2010.04.015 20430043 PMC2900526

[B170] Tayler K. K. Lowry E. Tanaka K. Levy B. Reijmers L. Mayford M. (2011). Characterization of NMDAR-independent learning in the hippocampus. *Front. Behav. Neurosci.* 5:28. 10.3389/fnbeh.2011.00028 21629769 PMC3099364

[B171] Teixeira C. M. Pomedli S. R. Maei H. R. Kee N. Frankland P. W. (2006). Involvement of the anterior cingulate cortex in the expression of remote spatial memory. *J. Neurosci.* 26 7555–7564. 10.1523/JNEUROSCI.1068-06.2006 16855083 PMC6674278

[B172] Teyler T. J. DiScenna P. (1986). The hippocampal memory indexing theory. *Behav. Neurosci.* 100 147–154. 10.1037/0735-7044.100.2.147 3008780

[B173] Teyler T. J. Rudy J. W. (2007). The hippocampal indexing theory and episodic memory: updating the index. *Hippocampus* 17 1158–1169. 10.1002/hipo.20350 17696170

[B174] Thome A. Marrone D. F. Ellmore T. M. Chawla M. K. Lipa P. Ramirez-Amaya V. (2017). Evidence for an evolutionarily conserved memory coding scheme in the mammalian hippocampus. *J. Neurosci.* 37 2795–2801. 10.1523/JNEUROSCI.3057-16.2017 28174334 PMC5354327

[B175] Tonegawa S. Liu X. Ramirez S. Redondo R. (2015a). Memory engram cells have come of age. *Neuron* 87 918–931. 10.1016/j.neuron.2015.08.002 26335640

[B176] Tonegawa S. Pignatelli M. Roy D. S. Ryan T. J. (2015b). Memory engram storage and retrieval. *Curr. Opin. Neurobiol.* 35 101–109. 10.1016/j.conb.2015.07.009 26280931

[B177] Trager S. C. Faber S. M. Dressler A. (2008). The stellar population histories of early-type galaxies – III. The Coma cluster. *Mon. Not. R. Astron. Soc.* 386 715–747. 10.1111/j.1365-2966.2008.13132.x 22538610

[B178] Tremblay P. Deschamps I. Baroni M. Hasson U. (2016). Neural sensitivity to syllable frequency and mutual information in speech perception and production. *Neuroimage* 136 106–121. 10.1016/j.neuroimage.2016.05.018 27184201

[B179] Treves A. (2009). “Spatial cognition, memory capacity and the evolution of mammalian hippocampal networks,” in *Cognitive Biology: Evolutionary and Developmental Perspectives on Mind, Brain and Behavior, Vienna Series in Theoretical Biology*, eds Tommasi L. Peterson M. A. Nadel L. (New York, NY: MIT Press), 41–59.

[B180] Treves A. (2016). “The dentate gyrus, defining a new memory of David Marr,” in *Computational Theories and their Implementation in the Brain: the Legacy of David Marr*, eds Vaina L. M. Passingham R. E. (Oxford: Oxford University Press).

[B181] Treves A. Rolls E. T. (1994). Computational analysis of the role of the hippocampus in memory. *Hippocampus* 4 374–391. 10.1002/hipo.450040319 7842058

[B182] Tulving E. (1972). “Episodic and semantic memory,” in *Organisation and Memory*, eds Tulving E. Donaldson W. (New York, NY: Academic Press), 382–403.

[B183] Ursano R. J. Goldenberg Z. Zhang L. Carlton J. Fullerton C. S. Li H. (2010). Posttraumatic stress disorder and traumatic stress: from bench to bedside, from war to disaster. *Ann. N. Y. Acad. Sci.* 1208 72–81. 10.1111/j.1749-6632.2010.05721 20955328

[B184] Walker M. P. Brakefield T. Hobson J. A. Stickgold R. (2003). Dissociable stages of human memory consolidation and reconsolidation. *Nature* 425 616–620. 10.1038/nature01930 14534587

[B185] Wang S. H. de Oliveira Alvares L. Nader K. (2009). Cellular and systems mechanisms of memory strength as a constraint on auditory fear reconsolidation. *Nat. Neurosci.* 12 905–912. 10.1038/nn.2350.19543280 19543280

[B186] Wartman B. C. Holahan M. R. (2013). The use of sequential hippocampal-dependent and -non-dependent tasks to study the activation profile of the anterior cingulate cortex during recent and remote memory tests. *Neurobiol. Learn. Mem.* 106 334–342. 10.1016/j.nlm.2013.08.011 23994429

[B187] Wartman B. C. Holahan M. R. (2014). The impact of multiple memory formation on dendritic complexity in the hippocampus and anterior cingulate cortex assessed at recent and remote time points. *Front. Behav. Neurosci.* 8:128. 10.3389/fnbeh.2014.00128 24795581 PMC4001003

[B188] Wiltgen B. J. Silva A. J. (2007). Memory for context becomes less specific with time. *Learn. Mem.* 14 313–317. 10.1101/lm.430907 17522020

[B189] Wiltgen B. J. Zhou M. Cai Y. Balaji J. Karlsson M. G. Parivash S. N. (2010). The hippocampus plays a selective role in the retrieval of detailed contextual memories. *Curr. Biol.* 20 1336–1344. 10.1016/j.cub.2010.06.068 20637623 PMC2928141

[B190] Winocur G. Moscovitch M. Bontempi B. (2010). Memory formation and long-term retention in humans and animals: convergence towards a transformation account of hippocampal-neocortical interactions. *Neuropsychologia* 48 2339–2356. 10.1016/j.neuropsychologia.2010.04.016 20430044

[B191] Winocur G. Moscovitch M. Sekeres M. (2007). Memory consolidation or transformation: context manipulation and hippocampal representations of memory. *Nat. Neurosci.* 10 555–557. 10.1038/nn1880 17396121

[B192] Winocur G. Moscovitch M. Sekeres M. J. (2013). Factors affecting graded and ungraded memory loss following hippocampal lesions. *Neurobiol. Learn. Mem.* 106 351–364. 10.1016/j.nlm.2013.10.001 24120426

[B193] Wong-Parodi G. Fischhoff B. Strauss B. (2015). Resilience vs. Adaptation: framing and action. *Clim. Risk Manag.* 10 1–7. 10.1016/j.crm.2015.07.002

[B194] Yassa M. A. Reagh Z. M. (2013). Competitive trace theory: a role for the hippocampus in contextual interference during retrieval. *Front. Behav. Neurosci.* 7:107. 10.3389/fnbeh.2013.00107 23964216 PMC3740479

